# Receptor engineering constitutes feedback control and robustness of IL-23R signaling and highlights importance of intracellular cytokine receptor signaling motifs

**DOI:** 10.1186/s12964-025-02593-2

**Published:** 2026-01-06

**Authors:** Leorina Kashtanjeva, Christin Ruhland, Franz Christian Horstmeier, Felix Thives-Kurenbach, Julia Ettich, Giacomo Padrini, Sophie Streuber, Jürgen Scheller, Anna Dittrich, Doreen M. Floss

**Affiliations:** 1 Institute of Biochemistry and Molecular Biology II, Medical Faculty and University Hospital Düsseldorf, Heinrich-Heine-University Düsseldorf, Düsseldorf, Germany; 2 Department of Systems Biology, Institute of Biology, Otto-von-Guericke-University Magdeburg, Magdeburg, Germany

**Keywords:** Signal transduction, IL-23, IL-6, Gp130, JAK-STAT, MAPK, SOCS3, Information theory, Mutual information

## Abstract

**Background:**

In the current medical landscape, synthetic cytokine receptors have emerged as a pivotal component in the development of novel therapeutic interventions. Interleukin-23 (IL-23) is the dominant regulatory cytokine in a cluster of immune-mediated inflammatory diseases induced due to the increased expression of the IL-23 receptor (IL-23R) on pathogenic TH17 cells. The modulation of IL-23 signaling by altering IL-23R presents a promising avenue for further investigation. Chimeric cytokine receptors expressing the extracellular domain (ECD) of one protein and the intracellular domain (ICD) of another have been used to isolate the effects of ligand binding from signaling.

**Methods:**

We designed chimeric IL-23Rs that comprise the extracellular and transmembrane domains of IL-23R, as well as various parts of the intracellular region of gp130, the IL-6 signal transducing receptor. To characterize signaling properties by these synthetic cytokine receptors analysis of the activation of the signaling proteins ERK1/2 and STAT3, and SOCS3 was combined with multiplexed single-cell flow cytometry data and information theoretic approaches.

**Results:**

Simply transferring the SOCS3-binding site from the IL-6 signal transducing receptor gp130 to IL-23R was not enough to make IL-23 signaling sensitive to SOCS3. However, an iterative transfer process identified a region of gp130 that bound SOCS3 and rendered IL-23 signaling sensitive to SOCS3 regulation. Opposingly, SOCS3-independent gp130 hyper-signaling was achieved by transferring a minimal IL-23R ERK activation motif to gp130. Notably, this motif retained gp130-dependent ERK activation without negative SOCS3 feedback.

**Conclusions:**

In summary, this study identifies the WLYEDIPN motif in IL-23R as an indispensable motif for IL-23R signal transduction and emphasizes the complexity of IL-23-induced signaling pathways and the function of various tyrosines in the intracellular part of cytokine/IL-23 receptor(s).

**Supplementary Information:**

The online version contains supplementary material available at 10.1186/s12964-025-02593-2.

## Abstract (plain language summary)

Cellular communication is facilitated by soluble molecules that transmit information from one cell to another. Cytokines are important signaling mediators in the immune system. They are released from sender cells and interact with their respective receptors on receiver cells inducing intracellular signaling cascades resulting in biological responses. Most cytokines bind to membrane spanning receptors that are linked to Janus kinases via their intracellular domain. Binding of a cytokine to the extracellular part of these receptors activates the JAK-STAT signaling pathway, which plays a role in maturation of blood cells, developing the immune system, growing and developing sexually, and turning fat cells into other types of cells. Any receptor that activates intracellular signaling pathways is equipped with negative feedback systems ensuring that the activated intracellular signaling pathways and downstream transcription factors are only activated temporarily. Suppressor of cytokine signaling (SOCS) proteins stop the action of many, but not all, JAK-STAT activating receptors. Here, we show that IL-23 signaling is insensitive to SOCS3 in contrast to IL-6 signaling. By changing the intracellular part of the IL-23R inside the cell we have created a novel artificial cytokine receptor that can be controlled by SOCS3. Additionally, we were able to show that a specific motif in IL-23R is important for its unique signal transduction. However, introducing this IL-23R motif in the IL-6 signal transducing receptor gp130 led to a SOCS3-insensitive receptor. In the future, we might be able to use receptors with modified signal transduction to treat certain diseases more effectively.

**Supplementary Information:**

The online version contains supplementary material available at 10.1186/s12964-025-02593-2.

## Background

Cytokines are humoral mediators that mediate cellular communication in the immune system. Cellular communication is facilitated by four distinct types of cytokine receptors that have been categorized by Yoshimura et al. according to their main signaling pathway: (a) receptors that activate nuclear factor (NF)-kB and mitogen-activated protein (MAP) kinases (mainly p38 and JNK); (b) receptors that activate the Janus kinase and signal transducers and activators of transcription (JAK-STAT) pathway; (c) transforming growth factor (TGF)-β receptors; and (d) the growth factor receptor family which typically induces activation of the MAPK ERK pathway [1]. It is evident that any receptor which activates intracellular signaling pathways is equipped with negative feedback systems, which ensure transient activation of the pathways and downstream transcription factors. Negative feedback plays a pivotal role in maintaining homeostasis because the absence of such negative regulators has been demonstrated to be a contributing factor to the development of various diseases [1]. The majority of cytokines bind to receptors with associated Janus kinases and induce the JAK-STAT signaling pathway, which is involved in hematopoiesis, immune development, sexual maturation, adipogenesis, and other important biological processes [2]. Accordingly, the negative regulation of this signaling pathway, which is achieved through the action of distinct mechanisms, is of paramount importance. These mechanisms include: protein tyrosine phosphatases (PTPs), protein inhibitor of activated STAT (PIAS), and suppressor of cytokine signaling (SOCS) proteins. The SOCS protein family consists of eight members, SOCS1 – SOCS7 and the cytokine-inducible Src homology 2 (SH2) containing protein (CIS), which inhibit STAT activation by many, but not all, JAK–STAT activating receptors [3]. SOCS proteins are structurally similar in that they all have an SH2 domain at their center and a SOCS box fragment at their C-terminus. However, the sequence at the amino terminus varies. A kinase inhibitory region (KIR) is present in SOCS1 and SOCS3 [4]. The expression of SOCS3 is induced by cytokines that activate STAT3 [5, 6]. SOCS3 is recruited to cytokine receptors via its SH2 domain to phosphorylated tyrosine motifs within the intracellular domain of these receptors, which allows SOCS3 to inhibit the activity of adjacent JAKs via the KIR domain [7]. However, SOCS3 can also mediate proteasomal degradation by assembling components of the E3 ubiquitin ligase complex [8, 9]. SOCS3 is the primary inhibitor of IL-6 signaling. This is mediated by the interaction between SOCS3 and pY759 (Y757 in mice) of gp130 [10], whereby SOCS3 binds simultaneously to the cytokine receptor and JAK [11]. Additionally, the constitutively expressed SH2-domain containing protein tyrosine phosphatase 2 (SHP2) binds via its SH2 domain to pY759 of gp130 [10, 12], gets phosphorylated by JAKs and functions as inhibitor of basal and cytokine-induced STAT3 activation [13–15]. Furthermore, binding of SHP2 to the pY759 motif in gp130, followed by phosphorylation by JAKs, initiates the activation of the MAPK pathway [16].

IL-23 is a member of the IL-12 cytokine family and consists of p19, a four-helix-bundle α subunit closely related to IL-6, and the β subunit p40 [17]. Signal transduction is mediated via the heterodimeric IL-23 receptor complex comprising IL-23R and IL-12Rβ1 [18]. The receptors lack intrinsic kinase activity and associate with Janus kinases (IL-23R: JAK2; IL-12Rβ1: TYK2) resulting in the activation of the JAK-STAT, MAPK and PI3K/Akt signaling pathways [19]. Due to the close relationship between IL-6 and p19, as well as gp130 and IL-23R, parallels in signal transduction were identified. Accordingly, the Y397EDI in IL-23R sequence was hypothesized as a potential SHP2 binding site [18, 20, 21]. In vitro studies of IL-23 signaling revealed that IL-23 triggered persistent STAT3 activation despite elevated SOCS3 expression. This finding suggests that IL-23R might not be a direct target of SOCS3 [21–23]. However, IL-23-induced STAT3 activation increased in T cells lacking SOCS3, and the loss of SOCS3 resulted in enhanced TH17 cell differentiation and cytokine expression [24]. It has been suggested that IL-23 stabilizes the TH17 phenotype by maintaining IL-17 production from fully differentiated effector cells and/or promoting their survival [25]. Consequently, IL-23 has been identified as a pro-inflammatory cytokine that plays a pivotal role in the development of chronic inflammatory diseases. This is attributed to its capacity to stimulate the production of inflammatory mediators, including IL-17, IL-22, GM-CSF, and TNF. These mediators, in turn, trigger the recruitment and activation of granulocytes and macrophages, which subsequently leads to tissue damage and chronic inflammation [26]. The discovery of the central role of the IL-23/IL-17 axis in autoimmune diseases opens up the possibility for new therapies [25, 27]. This includes inhibitors of the receptor-associated Janus kinases which target the action of the cytokine by blocking intracellular signaling pathways. Modification of cytokine signaling by altering cytokine receptors may represent an opportunity for new innovative genetic therapies. In the context of modification IL-23 signaling, the initiation of a negative feedback mechanism could constitute a viable approach to limit synthetic IL-23 signaling. Given that SOCS3 expression does not reduce IL-23-induced STAT3 activation, it is feasible to genetically modify the IL-23R to enable a negative feedback mechanism via SOCS3.

Here, we used the receptor gp130 as a template to make IL-23R signaling a target for SOCS3 feedback. The analysis of the engineered IL-23Rs was done in stably transduced Ba/F3-gp130 cells expressing the IL-12β1 and was focused on STAT3 and ERK activation. In addition to analyzing STAT3 phosphorylation using Western blot, we used multiplexed single-cell flow cytometry data and the information theoretic approach mutual information (MI) to characterize IL-23 signaling. In information theory transmission of a signal from a sender (input) to a receiver (output) via noisy channel is analyzed. This concept can easily be transferred to signaling pathways where information from an activated receptor is transmitted to an activated transcription factor through a channel that is affected by stochasticity of biochemical reactions and cell-to-cell heterogeneity. However, also any two other signaling proteins within a signaling pathway can be defined as input and output respectively. MI is a measure of correlation between input and output that allows dealing with non-linear effects inherent to biological processes [28]. MI can also be used to quantify robustness of STAT3 activation against cell-to-cell variability. Robust phosphorylation of STAT3 (output) depends on the presence of a cytokine but is independent of variation in STAT3 protein copy number (input). We have recently shown, that negative feedback by SOCS3 increases robustness of IL-6-induced STAT3 activation against variability in STAT3 expression [29]. Our findings demonstrated that in contrast to IL-6-induced STAT3 activation IL-23-induced STAT3 activation is resistant to negative feedback from SOCS3 and thus also robustness of IL-23 signaling was independent of SOCS3. Engineering of synthetic IL-23Rs that render IL-23 signaling responsive to negative feedback by SOCS3 also ensured robustness of STAT3 activation. Furthermore, the engineering of IL-23R yielded critical insights into the complex nature of IL-23 signal transduction.

## Methods

### Cells and reagents

Ba/F3-gp130 cells were previously described in another publication [30]. Phoenix-Eco cells were obtained from Ursula Klingmüller (DKFZ, Heidelberg, Germany). HEK293T (ACC 635) cells were purchased from the Leibnitz Institute DSMZ-German Collection of Microorganisms and Cell Culture (Braunschweig, Germany). Dulbecco’s modified Eagle medium with high glucose concentration (GIBCO^®^, Thermo Fisher Scientific, Waltham, MA, USA) containing 10% fetal bovine serum (GIBCO^®^), 60 mg/l penicillin, and 100 mg/l streptomycin (Genaxxon bioscience GmbH, Ulm, Germany) was utilized to cultivate cell lines at 37 °C with 5% CO₂. The cultivation of Ba/F3-gp130 cells was performed using Hyper-IL-6 (HIL-6), a product derived from the conditioned cell culture medium of HIL-6-secreting CHO-K1 cells or from a recombinant source, namely ExpiCHO-S™ cells (obtained from Thermo Fisher Scientific). HIL-6 is a fusion protein composed of IL-6 and the soluble IL-6R, which are connected via a flexible peptide linker [31]. Hyper-IL-23 (HIL-23) is a fusion protein composed of p40 and p19, linked by a flexible peptide linker [17]. Human HIL-23 and HIL-6 were expressed with Twin-Strep-tag^®^ (iba GmbH, Göttingen) and subsequently purified as previously described [32, 33].

The following antibodies were obtained from Cell Signaling Technology (Leiden, The Netherlands); phospho-STAT3 ((Tyr705) (D3A7) #9145), STAT3 ((79D7) #4904), phospho-p44/42 MAPK (Erk 1/2) (Thr202/Tyr204) (#4370), p44/42 MAPK (Erk 1/2) antibody (#9102), phospho-Jak2 (Tyr1007/1008) (#3771) and Jak2 ((D2E12) #3230). Goat anti-rabbit IgG (H + L) secondary antibody, peroxidase-conjugated (#31462) and rabbit anti-goat IgG (H + L) secondary antibody, peroxidase-conjugated (#31402) were obtained from Thermo Fisher Scientific. Furthermore, human IL-23R biotinylated antibody (#BAF1400, R&D Systems, Minneapolis, MN, USA), Alexa Fluor 647–conjugated Fab goat anti-rat IgG (#112–607−003, Dianova, BIOZOL Diagnostica Vertrieb GmbH, Hamburg, Germany), anti-hIL-12Rβ1 PE conjugated antibody (#FAB839, R&D Systems), CIS3/SOCS-3 anti-human rabbit IgG (#C005, IBL America, Minneapolis, MN, USA), PE mouse anti-STAT3 (#560391, BD Biosciences, Franklin Lakes, NJ, USA) and Alexa Fluor 647 mouse anti-(p)STAT3 (pY705) (#557815, BD Biosciences) were used. Anti-FLAG^®^ antibody (#7425) and ANTI-FLAG^®^ M2 affinity gel (#A2220) were purchased from Merck (Darmstadt, Germany).

### Cloning

The cloning of the wild-type human IL-23R has been described previously [34]. IL-23R deletion variants with truncated intracellular domains were cloned using standard PCR. IL-23R variants with SOCS3/SHP2 binding motif insertions from gp130 were generated using splicing by overlap extension (SOE) PCR and inserted into the p409 expression vector [35]. Valine insertion was generated by PCR using Phusion high-fidelity DNA polymerase, followed by DpnI digestion of methylated template DNA [36]. The synthetic IL-23R_ET_gp130_I_ chimera contains the coding sequences for the human IL-23R signal peptide (SP, Q5VWK5, M1-G23), followed by the sequences for the extracellular domain (G24-G355) and the transmembrane domain (L356-F376) of IL-23R, and the cytoplasmic domain of human gp130 (P40189, N642-Q918). The IL-23R_ET_gp130_I_ variant with the insertion of the SHP2-binding motif from IL-23R (WLYEDIPN) was generated using SOE-PCR. Expression plasmids for the synthetic IL-23R_ET_gp130_I_ variants were designed by combining the extracellular and transmembrane domain coding sequences of IL-23R with the gp130 cytosolic domain coding sequences (N642-S765 or N642-Q813), resulting in the IL-23R_ET_gp130_I(N642−S765)_-IL-23R_I(G483−K629)_ and IL-23R_ET_gp130_I(N642−Q813)_-IL-23R_I(G483−K629)_ variants. To generate the Y767F gp130 variant, an expression vector containing the cDNA for the synthetic receptor was used as a template for site-directed mutagenesis. The resulting expression cassettes were transferred into the retroviral vector pMOWS-puro [37], which was then used to transduce Ba/F3-gp130 cells that express human IL-12Rβ1 [34]. The coding sequence for human SOCS3 (O14543, M1-L225) was cloned with a C-terminal FLAG tag and a substitution of the PEST motif (P129-R163) with a (GS)₄ linker [38]. All generated expression plasmids were verified by sequencing.

### Transduction and transfection of cells

The Ba/F3-gp130-IL-12Rβ1 cells were retrovirally transduced with pMOWS expression plasmids that code for the IL-23R variants, as previously described by Franke et al. [34]. The transduced Ba/F3 cells were selected using 1 mg/ml hygromycin B and 1.5 µg/ml puromycin (Carl Roth GmbH, Karlsruhe, Germany) for at least two weeks. The generated Ba/F3-gp130 cell lines were then analyzed for synthetic receptor cell surface expression via flow cytometry. HEK293T cells (2 × 10^6^) were transiently transfected as indicated using TurboFect transfection reagent (Fermentas, Thermo Scientific) according to the manufacturer’s instructions.

### Cell surface detection of cytokine receptors via flow cytometry

To detect cell surface expression of cytokine receptors, stably transduced Ba/F3 cells were washed with FACS buffer (PBS containing 1% BSA) and incubated with 5 × 10⁵ cells/50 µl of FACS buffer supplemented with antibodies against human IL-12Rβ1 or IL-23R for 1 h. After a single wash with FACS buffer, the cells were incubated in 50 µl of FACS buffer containing fluorophore conjugated secondary antibody for 1 h. Finally, the cells were washed with FACS buffer and analyzed by flow cytometry using a BD FACSCanto II flow cytometer and FACSDiva software (BD Biosciences). The data were analyzed using FCS Express software (De Novo Software, Los Angeles, CA, USA).

### Cell viability assay

Ba/F3-gp130 cells were washed three times and adjusted to 5 × 10^3^ cells in 100 µl DMEM supplemented with 10% FCS, 60 mg/l penicillin, and 100 mg/l streptomycin. Ligands were added, and cells were incubated for 3 days at 37 °C with 5% CO₂. All values were measured in triplicates per experiment. 20 µl of CellTiter-Blue^®^ Reagent (Promega, Madison, WI, USA) was added per well and fluorescence was immediately measured with the Infinite M200 PRO plate reader (Tecan, Crailsheim, Germany) to determine the cell viability (emission 590 nm, excitation 560 nm). These measurements were made every 20 min for 2 h. The fluorescent signal from the CellTiter-Blue^®^ Reagent is directly proportional to the number of viable cells. All values were measured in triplicates per experiment, and the fluorescence values were normalized by subtraction of the time point 0 values. Experiments were performed at least three times, and one representative experiment was selected for presentation. Data are presented as means ± SD. For the multiple comparisons, two-way ANOVA and Bonferroni correction were used in GraphPad Prism 8. Statistical significance was set to *p* ≤ 0.05 (*p ≤ 0.05, **p ≤ 0.01, ***p ≤ 0.001).

### Stimulation of cells and analysis of intracellular signal transduction by Western blot

The various Ba/F3-gp130-IL-12Rβ1-IL-23R cell lines were washed three times with PBS and subsequently starved in serum-free medium for 4 h. The cells were then stimulated with the indicated ligands up to 6 h, harvested by centrifugation at 4 °C for 5 min at 1500 rpm, and frozen. The cells were lysed for two hours using a buffer containing 10 mM Tris–HCl (pH 7.8), 150 mM NaCl, 0.5 mM EDTA, 0.5% Nonidet P-40, 1 mM sodium vanadate, 10 mM MgCl₂, and one complete EDTA-free protease inhibitor tablet (Roche Diagnostics). The protein concentration of the cell lysates was determined using the bicinchoninic acid protein assay (Pierce, Thermo Fisher Scientific). The lysed proteins were then mixed with (5x) SDS loading buffer (125 mM Tris-HCl pH 6.8, 50% glycerol, 10% SDS, 5% β-mercaptoethanol, bromophenol blue) and incubated at 95 °C for 10 min. The separation of proteins was conducted via SDS-PAGE, followed by their transfer to polyvinylidene difluoride (PVDF) membranes for 60 min (20 V, 1 A). The membranes were then blocked in 5% fat-free dried skimmed milk in TBS-T (10 mM Tris HCl pH 7.6, 150 mM NaCl, 1% Tween 20) and probed overnight with the indicated primary antibodies (1:1000). After washing, the membranes were incubated with secondary peroxidase–conjugated antibodies (1:2000). The Immobilon Western Reagents (Millipore Corporation) and the ChemoCam Imager (INTAS Science Imaging Instruments GmbH) were used for signal detection.

### Intracellular staining of pSTAT3 and STAT3

To analyze intracellular STAT3 phosphorylation Ba/F3 cells were washed with PBS and starved for 3 h in DMEM without FCS and antibiotics. After stimulation with HIL-6 or HIL-23 as indicated, cells were fixed. Therefore, 100 µl of the cell suspension was mixed with 100 µl paraformaldehyde (4%) and incubated at 37 °C for 10 min followed by centrifugation at 300 *g*, 4 °C for 5 min. Cell pellets were suspended in ice cold 90% methanol and incubated at −20 °C for 10 min. Subsequently, cells were washed twice with ice-cold BSA-EDTA buffer (1% BSA, 2 mM EDTA in PBS) and incubated with saturated fluorophore-coupled primary antibodies against STAT3 and pSTAT3 overnight. Cells were washed again for three times in 1% BSA-EDTA buffer before FACS analysis. Cells were analyzed by flow cytometry using a BD FACSCanto II flow cytometer and FACSDiva software (BD Biosciences). Data were evaluated using FlowJo (version 10.6.1., BD Biosciences).

### Mutual information (MI)

MI is a measure of statistical dependence between any two variables that is robust to nonlinearity, thereby outperforming classical correlation. We use MI as a measure for robustness of STAT3 activation against heterogenous expression of STAT3. Robust STAT3 activation depends on the given cytokine stimulus, but is independent of variation in protein copy number of STAT3 between individual cells. MI was computed by the formula [39]:

$$\:MI(S;R)=\iint\:p(S,R){\mathrm{log}}_{2}\frac{p(S,R)}{p\left(S\right)p\left(R\right)}dRdS$$

*R* and *S* denote the two variables to be analyzed, in our case the extent of STAT3 phosphorylation and STAT3 expression, respectively. *p(S*,* R)* is the joint probability density of these two variables and *p(S)* and *p(R)* are the marginal probability densities of the respective variables. The computation was done with a custom-made Python script [15, 29] using the statistics and integration modules in the “scipy” package (version 0.15.1, Enthought, Austin, TX, USA). All probability densities were approximated by kernel density estimation [40] and the integration was performed with the “quadpack” library. As input data results from multiplexed flow cytometric analyses of cytokine-dependent STAT3 Y705 phosphorylation and STAT3 expression were used.

### Co-immunoprecipitation analysis

For co-immunoprecipitation (co-IP) using an ANTI-FLAG^®^ M2 affinity gel (Merck), HEK293T cells were transiently transfected with cDNAs encoding the IL-23R variants and SOCS3. Forty-eight hours after transfection, the cells were lysed in a solution containing 50 mM Tris-HCl (pH 7.5), 1 mM EDTA, 150 mM NaCl and one complete protease inhibitor mixture tablet per 50 ml of buffer solution (Roche Diagnostics, Basel, Switzerland), supplemented with 1% Triton X-100, and left to stand on ice for 1 h. For the negative control, the IL-23R cDNAs were transfected without the SOCS3 cDNA. A total of 30 µl of ANTI-FLAG^®^ M2 affinity gel was then added and the mixture was incubated overnight at 4 °C under gentle agitation. The samples were then washed three times in the aforementioned buffer, excluding the 1% Triton X-100. Proteins were eluted by adding 50 µl of 2.5× Laemmli buffer and incubated for 10 min at 95 °C. The resulting supernatants were subjected to Western blot analysis.

### RT-PCR analyses

RNA purification was performed with the NucleoSpin RNA kit according to the manufacturer’s instructions (Macherey-Nagel, Düren, Germany). SOCS3 gene expression was analyzed using the iTaq Universal SYBR Green One-Step Kit (#1725151, Bio-Rad Laboratories Inc., Hercules, CA, USA). The expression level of SOCS3 was normalized to the expression of GAPDH for analysis (Δ cycle threshold). Gene expression values were calculated using the ΔΔ^Ct^ method. Gene expression was determined by 2^−ΔΔCt^.

## Results

### IL-23 signaling is not sensitive to SOCS3

**Fig. 1:**
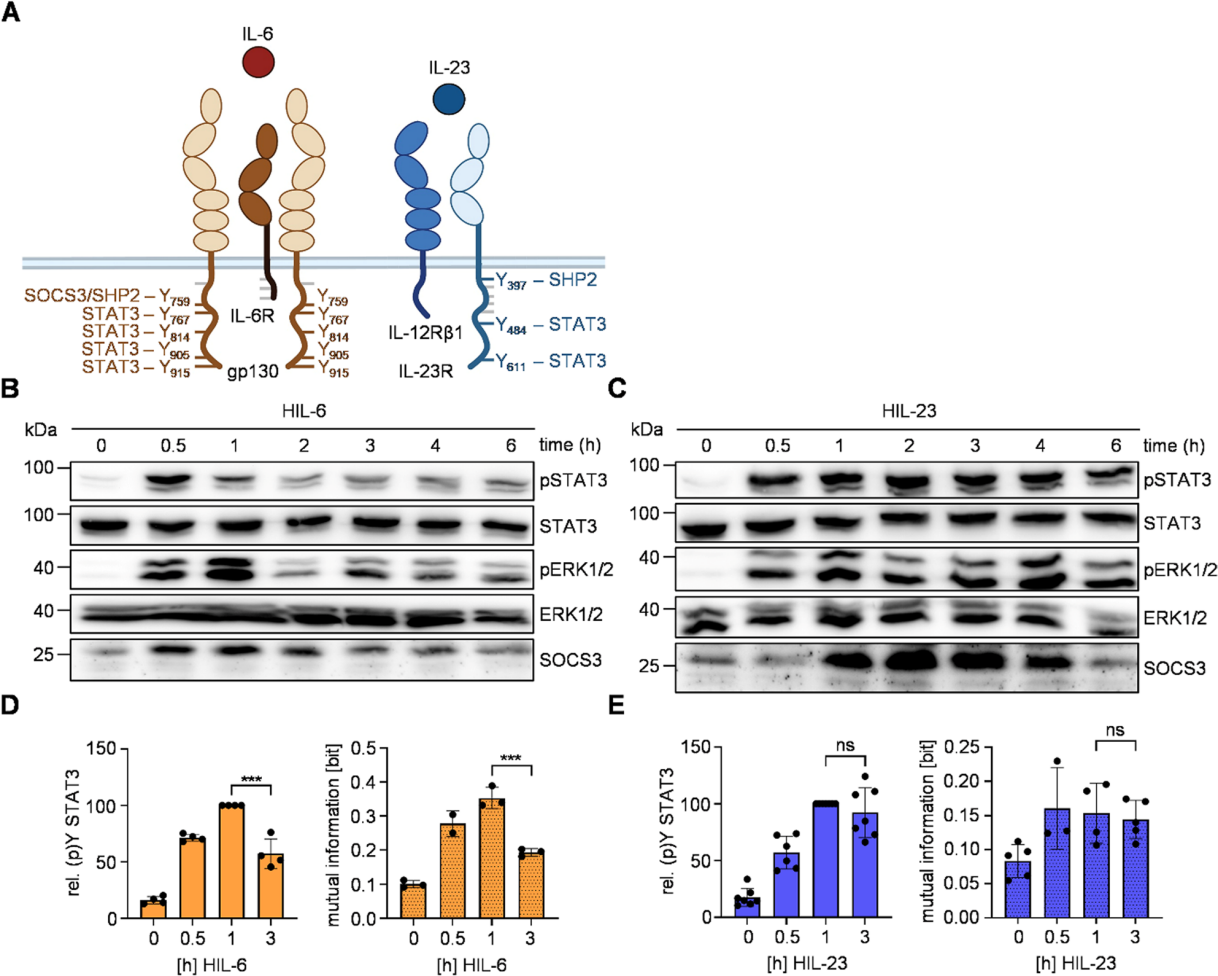
The IL-23 receptor complex is no target of SOCS3.** A** Schematic illustration of IL-6 and IL-23 receptor complexes. Tyrosines implicated in signal transduction pathways are shown with respective signaling proteins. **B + C** STAT3 and ERK1/2 activation in Ba/F3-gp130 cells expressing IL-12Rβ1 and IL-23R treated with HIL-6 (10 ng/ml) (**B**) or HIL-23 (10 ng/ml) (**C**) for indicated time points or left untreated. Equal amounts of proteins (50 µg/lane) were analyzed via specific antibodies detecting phospho-STAT3 and STAT3, phospho-ERK1/2 and ERK1/2, and SOCS3. Western blot data shows one representative experiment out of three. **D** + **E** Ba/F3-gp130-IL-12Rβ1-IL-23R cells were stimulated with HIL-6 (**D**) or HIL-23 (**E**) for the specified times. STAT3 expression (not shown) and STAT3-Y705 phosphorylation were assessed in parallel by multiplexed intracellular flow cytometry using fluorophore-coupled antibodies. For each independent time-point, the mean fluorescence intensity (MFI) of STAT3-Y705 staining was calculated from the single-cell data. The maximal MFI per experiment was normalized to 100%. Mutual information (MI) between STAT3 expression (not shown) and STAT3-Y705 phosphorylation was calculated from raw flow cytometry data in Ba/F3-gp130-IL-12Rβ1-IL-23R cells stimulated with HIL-6 or HIL-23 for the indicated times. Data represent mean ± SD from *n* = 4 (**D**) or *n* = 7 (**E**) experiments. Statistical analysis used one-way ANOVA, followed by Tukey correction, ***p ≤ 0.001, ns not significant

IL-6 and IL-23 signaling is mediated by receptor-associated Janus kinases that phosphorylate tyrosines within the cytoplasmic regions of the respective cytokine receptors (Fig. 1A). The four most membrane-distal tyrosines of human gp130 (Y767, Y814, Y905, Y915) play a critical role in the recruitment of STAT3. Y905 and Y915 have also been observed to facilitate the recruitment of STAT1. Tyrosine Y759 recruits SHP2, as well as the negative feedback inhibitor SOCS3 [10, 12, 13]. Y484 and Y611 within human IL-23R mediate STAT3 phosphorylation, whereas Y397 is involved in activation of PI3K/Akt and MAPK pathways via SHP2. Furthermore, a non-canonical site C-terminal to amino acid residue 476 but distinct from Y484 and Y611 in IL-23R is involved in STAT3 phosphorylation [21]. Ba/F3-gp130 cells expressing human IL-12Rβ1 and human IL-23R have been generated ([34], Additional Fig. 1A) and were stimulated with Hyper-IL-6 (HIL-6), a fusion protein of the soluble IL-6R and IL-6 connected via a flexible linker, to induce IL-6 signal transduction [31]. Alternatively, Ba/F3-gp130-IL-12Rβ1-IL-23R cells were treated with a fusion protein of p40 and p19, named Hyper-IL-23 (HIL-23) to induce IL-23 signaling and proliferation [17]. A comparative analysis of the kinetics exhibited by pSTAT3 and pERK1/2 following stimulation with either HIL-6 or HIL-23 revealed significant disparities. Stimulation with HIL-6 resulted in transient phosphorylation of both STAT3 and ERK1/2 accompanied by expression of the negative feedback inhibitor SOCS3 (Fig. 1B). In contrast, stimulation with HIL-23 resulted in sustained STAT3 and ERK1/2 phosphorylation (Fig. 1C), a phenomenon which has previously been demonstrated for signaling of murine and synthetic IL-23 receptors [21, 22]. Notably, also HIL-23 induced the expression of SOCS3 protein and mRNA, even to a higher level than HIL-6 (Fig. 1C, Additional Fig. 1B). Notably, SOCS3 interacts with the cytoplasmic region of IL-23R (Additional Fig. 2A). Nevertheless, IL-23 signaling is not inhibited by SOCS3.

Truncation of the IL-23R revealed that the SOCS3 binding site in IL-23R is probably located between amino acids L428 and Q555 (Additional Fig. 2B). Both HIL-23 and HIL-6 stimulation induce endogenous expression of both SOCS3 protein isoforms (Fig. 1B + C [41]). SOCS3 not only acts as negative feedback inhibitor of JAK-STAT signaling but also ensures robustness of IL-6-induced STAT3 activation against cell-to-cell heterogeneity in STAT3 expression level at late time-points [29]. As IL-23-induced STAT3 signaling is insensitive to SOCS3 feedback (Fig. 1C), we hypothesized that SOCS3 is also not involved in ensuring robustness of late IL-23 signaling. As readout of robustness, we calculated mutual information (MI) between STAT3 and phosphorylated STAT3 [15, 29]. High MI describes high dependency of STAT3 phosphorylation on STAT3 expression level and thus low robustness of STAT3 activation. STAT3 expression and phosphorylation were analyzed based on multiplexed single-cell flow cytometry data. Again, we show rapid HIL-6-induced STAT3 phosphorylation, reduced by SOCS3 induction in later phases of signaling. In line with previous results, stimulation of Ba/F3-gp130-IL-12Rβ1-IL-23R cells with HIL-6 reduced robustness of STAT3 phosphorylation as shown by increased MI at early time points, while expression of SOCS3 ensures robustness of HIL-6-induced STAT3 phosphorylation at late time points (Fig. 1D) [29]. As expected, HIL-23, like HIL-6 reduces robustness of early STAT3 phosphorylation. However, in contrast to HIL-6, HIL-23-induced signaling is not sensitive to negative feedback regulation by SOCS3 and consequently MI is not reduced over time (Fig. 1E).

In summary, IL-23-induced STAT3 activation is insensitive to negative feedback regulation by SOCS3 in Ba/F3 cells. Although IL-23 induces the expression of SOCS3, SOCS3 does not reduce IL-23-induced STAT3 phosphorylation and thus does not ensure robustness of late STAT3 activation against cell-to-cell heterogeneity in STAT3 expression.

### The insertion of the gp130 SOCS3 binding motif at Y397 of IL-23R does not result in the inhibition of IL-23 signaling through negative feedback

**Fig. 2:**
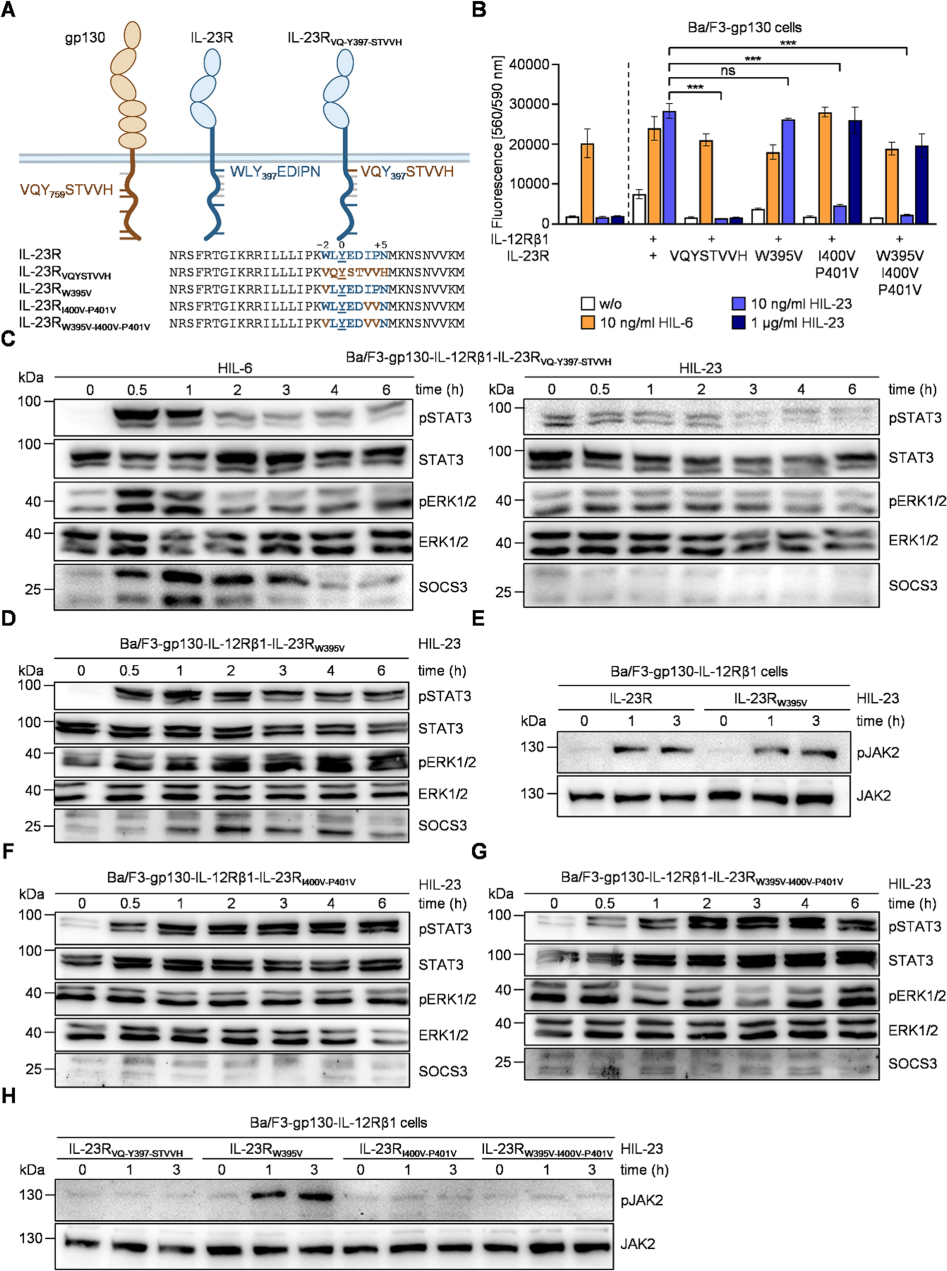
Amino acids surrounding Y397 in IL-23R are necessary for signal transduction and cell proliferation.** A** Schematic illustration of gp130 and IL-23R variants with modifications of the WLY_397_EDIPN motif. **B** Proliferation of Ba/F3-gp130 cells expressing IL-12Rβ1 and IL-23R variants without cytokine (w/o), with HIL-6 (10 ng/ml) and HIL-23 (10 ng/ml, 1 µg/ml). Error bars, SD. One representative experiment out of three is shown. Statistical analysis used two-way ANOVA, followed by Bonferroni correction, ***p ≤ 0.001, ns not significant. **C** STAT3 and ERK1/2 activation in Ba/F3-gp130 cells expressing IL-12Rβ1 and IL-23R_VQ−Y397−STVVH_ treated with HIL-6 (10 ng/ml) and HIL-23 (10 ng/ml) for indicated time points or left untreated. Equal amounts of proteins (50 µg/lane) were analyzed via specific antibodies detecting phospho-STAT3 and STAT3, phospho-ERK1/2 and ERK1/2, and SOCS3. Western blot data shows one representative experiment out of three. **D** STAT3 and ERK1/2 activation in Ba/F3-gp130 cells expressing IL-12Rβ1 and IL-23R_W395V_ treated with HIL-23 (10 ng/ml) for indicated time points or left untreated. Analysis was performed as described in C. Western blot data shows one representative experiment out of three. **E** JAK2 activation in Ba/F3-gp130 cells expressing IL-12Rβ1 and IL-23R or IL-23R_W395V_ treated with HIL-23 (10 ng/ml) for indicated time points or left untreated. Equal amounts of proteins (50 µg/lane) were analyzed via specific antibodies detecting phospho-JAK2 and JAK2. Western blot data shows one representative experiment out of three. **F + G** STAT3 and ERK1/2 activation in Ba/F3-gp130 cells expressing IL-12Rβ1 and IL-23R_I400V−P401V_ (**F**) or IL-23R_W395V−I400V−P401V_ (**G**) treated with HIL-23 (10 ng/ml) for indicated time points or left untreated. Analysis was performed as described in C. Western blot data shows one representative experiment out of three. **H** JAK2 activation in Ba/F3-gp130 cells expressing IL-12Rβ1 and IL-23R_VQ−Y397−STVVH_, IL-23R_W395V_, IL-23R_I400V−P401V_ or IL-23R_W395V−I400V−P401V_ treated with HIL-23 (10 ng/ml) for indicated time points or left untreated. Analysis was performed as described in E. Western blot data shows one representative experiment out of three

SOCS3 and SHP2 bind to a motif centered around phosphorylated Y759 in gp130 via their SH2 domains (VQpYSTVVH [42, 43]). Here, the side chains of pY - 1 and − 2 residues in addition to pY + 3, + 4, and + 5 interact with SOCS3 [44]. Analyzing SOCS3 interaction motifs within different cytokine receptors resulted in a minimal consensus motif of V/L-X-pY-X-X-V/L-V/L-X [45]. First of all, we asked whether the simple transfer/replacement of the SOCS3/SHP2-binding site from gp130 (VQ-Y759-STVHH) to the SHP2-only binding site of IL-23R (WL-Y397-EDIPN) renders IL-23R signaling a target for SOCS3 feedback. Cells expressing this synthetic IL-23R, named IL-23R_VQ−Y397−STVVH_ failed to proliferate in response to HIL-23 (Fig. 2A + B), although the receptor was expressed on the surface of Ba/F3-gp130 cells together with IL-12Rβ1 (Additional Fig. 3). This suggests that the IL-23R_VQ−Y397−STVVH_ itself is signaling incompetent. Indeed, HIL-23-induced ERK1/2 phosphorylation but also STAT3 phosphorylation was completely abrogated by transfer of the SOCS3/SHP2 site from gp130 to IL-23R at the position of the native SHP2 binding site in IL-23R_VQ−Y397−STVVH_ (Fig. 2C).

Therefore, we took an alternative approach and attempted to convert the SHP2 binding site in IL-23R into a SOCS3/SHP2 binding site by the stepwise introduction of specificity determining valine amino acid residues at positions pY - 2 (W395), pY + 3 (I400) and pY + 4 (P401) [42]. We generated three IL-23R variants IL-23R_W395V_, IL-23R_I400V−P401V_, and IL-23R_W395V−I400V−P401V_, which were expressed on the cell surface after stable transduction into Ba/F3-gp130-IL-12Rβ1 cells (Additional Fig. 3).

The capacity for IL-23-dependent proliferation decreased significantly with increasing number of inserted valines. Whereas W395V insertion into IL-23R was comparable to wild-type receptor, only high concentrations of HIL-23 induced proliferation of Ba/F3-gp130-IL-12Rβ1 cells expressing IL-23R with valine at position pY + 3 and + 4. HIL-6-dependent proliferation of the different Ba/F3 cell lines was not altered by expression of mutated IL-23 receptor variants (Fig. 2B). Analysis of signaling with focus on STAT3 and ERK1/2 activation supported these results. HIL-6-induced STAT3 and ERK1/2 phosphorylation was transient in all four Ba/F3-gp130-IL-12Rβ1-IL-23R cell lines accompanied by expression of SOCS3 (Additional Fig. 4). Ba/F3-gp130-IL-12Rβ1-IL-23R_W395V_ cells showed sustained HIL-23-induced STAT3 and ERK phosphorylation even though SOCS3 was expressed, comparably to cells expressing the wild-type IL-23R (Fig. 2D). This also applies to the activation of IL-23R-associated Janus kinase JAK2 (Fig. 2E). Insertion of valines at position 400 and 401 induced a delay of maximal HIL-23-induced activation of JAK-STAT pathway and a lower expression of SOCS3 compared to signaling via wild-type IL-23R (Fig. 2F + G). HIL-23 did not increase basal ERK1/2 phosphorylation in Ba/F3 cells expressing these variants of the IL-23R with multiple mutations in the SHP2 interaction site (Fig. 2F + G). We already demonstrated that MAPK activation is needed and sufficient for IL-23 dependent cellular proliferation of Ba/F3 cells [21]. Thus, the described alterations in ERK activation might be causative for the diminished cell proliferation at low cytokine concentrations (Fig. 2B). In the murine system, modification of the predicted SHP2 binding site WLYEDIPN in IL-23R (WL-F416-EDIPN) resulted in decreased JAK2, STAT3, and ERK1/2 activation [46]. Analogous outcomes were observed through the incorporation of valines at the pY + 3 and + 4 positions of human IL-23R. The Ba/F3-gp130-IL-12Rβ1 cells that express IL-23R_VQ−Y397−STVVH_, IL-23R_I400V−P401V_, or IL-23R_W395V−I400V−P401V_ demonstrated no activation of JAK2 when stimulated with HIL-23 (Fig. 2H). Therefore, it can be deduced that the activation of JAK2 is influenced by the insertion of valines within the human IL-23R near the putative SHP2 binding side Y397. Consequently, this results in a reduced activation of STAT3 and remarkably low expression of SOCS3. Additionally, reduced JAK2 activation reduces SHP2 phosphorylation, which prevents activation of the IL-23-induced MAPK signaling pathway (Fig. 2F + G).

**Fig. 3:**
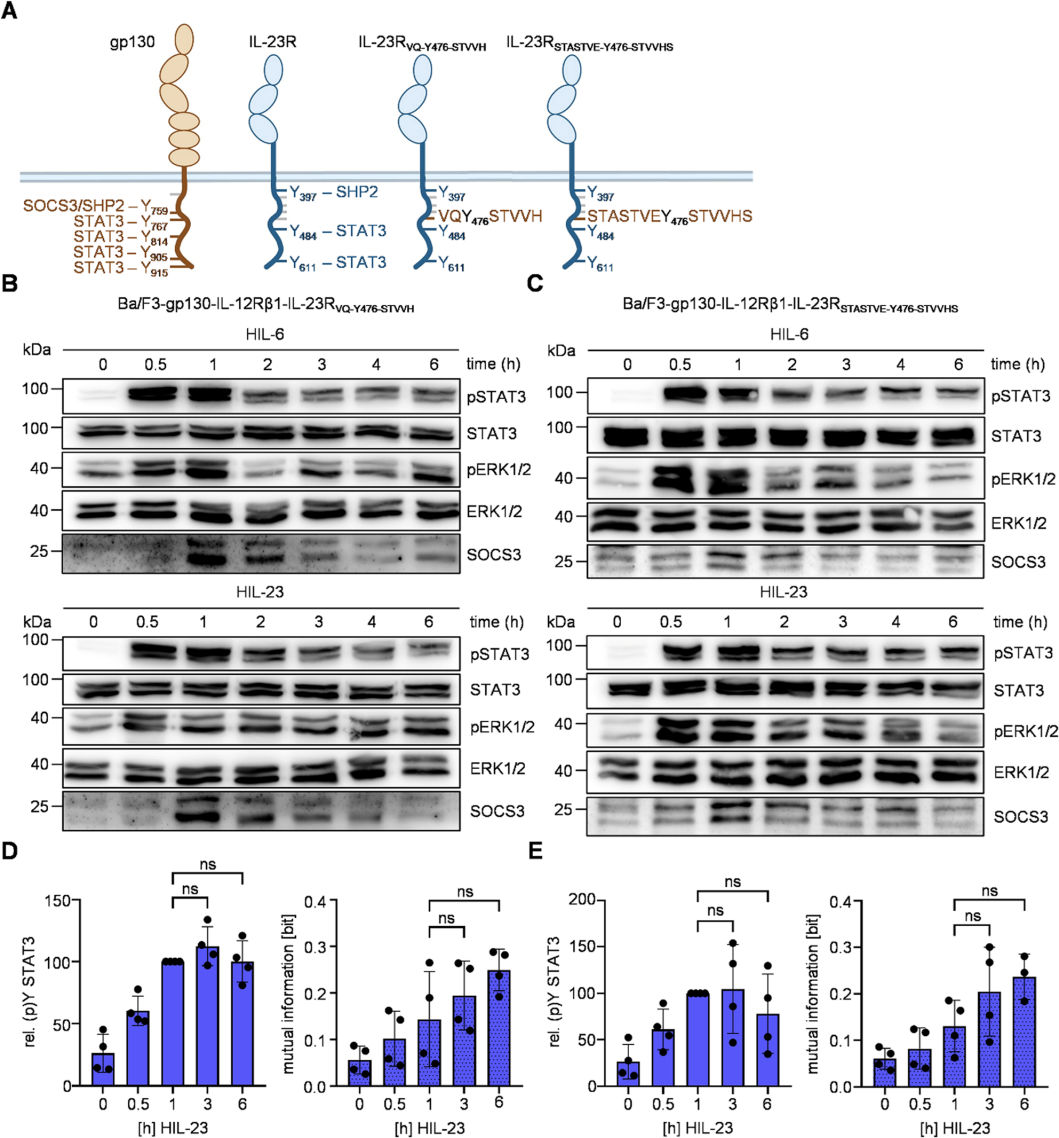
Insertion of the gp130 SOCS3 binding motif in IL-23R around Y476 induces no negative feedback.** A** Schematic illustration of gp130, IL-23R and modified variants. Tyrosines implicated in signal transduction pathways are shown with respective signaling proteins, gp130 binding motifs are highlighted in brown. **B + C** STAT3 and ERK1/2 activation in Ba/F3-gp130 cells expressing IL-12Rβ1 and IL-23R_VQ−Y476−STVVH_ (**B**) or IL-23R_STASTVE−Y476−STVVHS_ (**C**) treated with HIL-6 (10 ng/ml) or HIL-23 (10 ng/ml) for indicated time points or left untreated. Equal amounts of proteins (50 µg/lane) were analyzed via specific antibodies detecting phospho-STAT3 and STAT3, phospho-ERK1/2 and ERK1/2, and SOCS3. Western blot data shows one representative experiment out of three. **D** + **E** Ba/F3-gp130-IL-12Rβ1-IL-23R_VQ−Y476−STVVH_ (**D**) or Ba/F3-gp130-IL-12Rβ1-IL-23R_STASTVE−Y476−STVVHS_ (**E**) cells were stimulated with HIL-23 for the specified times. STAT3 expression (not shown) and STAT3-Y705 phosphorylation were assessed in parallel by multiplexed intracellular flow cytometry using fluorophore-coupled antibodies. For each independent time-point, the mean fluorescence intensity (MFI) of STAT3-Y705 staining was calculated from the single-cell data. The maximal MFI per experiment was normalized to 100%. Mutual information (MI) between STAT3 expression (not shown) and STAT3-Y705 phosphorylation was calculated from raw flow cytometry data in respective Ba/F3 cells stimulated with HIL-23 for the indicated times. Data represent mean ± SD from *n* = 4 experiments. Statistical analysis used one-way ANOVA, followed by Tukey correction, ns not significant

The straightforward transfer of the SOCS3/SHP2 binding site (VQYSTVHH) from gp130 to IL-23R proved ineffective in inducing negative feedback inhibition by SOCS3. Consequently, an alternative approach was devised, taking into account the distinct organization of the intracellular domains of gp130 and IL-23R. Y759 of gp130 is more distant (117 aa) from the transmembrane domain than Y397 of IL-23R (20 aa), and C-terminal of the Box1/Box2 motifs in gp130, which are important for the association with JAKs [47, 48]. The interaction of JAK2 with IL-23R is not mediated by the classical Box1/Box2 motifs. Instead, the amino acid sequence E455 to E479 (human: E436-459) in IL-23R is important but not sufficient for JAK2 activation in mouse cells [46]. In addition, the motif surrounding Y397 in IL-23R appears to be a critical element in the process of JAK2 activation (Fig. 2H). To prevent interference of the engineered SOCS3 binding side with JAK activation, we thus next transferred the human gp130 SOCS3/SHP2 binding motif VQYSTVVH to Y476 of IL-23R which is 99 aa C-terminal of the transmembrane domain and not involved in signal transduction [21]. In addition, the longer STASTVEYSTVVHS SOCS3/SHP2 binding motif of murine gp130 (Fig. 3A) was inserted around Y476 of IL-23R [11]. The resulting Ba/F3-gp130-IL-12Rβ1 cell lines (Additional Fig. 5) exhibited transient STAT3 and ERK activation following HIL-6 stimulation due to expression of the negative feedback inhibitor SOCS3 (Fig. 3B + C, Additional Fig. 6A). Stimulation of these cells with HIL-23 induced rapid phosphorylation of STAT3 and ERK1/2 which is slightly reduced at late time points when analyzed by Western blot (Fig. 3B + C). The kinetics of HIL-23-induced STAT3 phosphorylation were supported by intracellular staining of pSTAT3 through a slight decline in late STAT3 phosphorylation that however did not reach statistical significance (Fig. 3D + E). In line with low responsiveness to SOCS3 feedback, robustness of late STAT3 phosphorylation is not ensured in Ba/F3 cells expressing IL-23 receptors containing the human or murine SOCS3/SHP2 binding side around Y476 as indicated by sustained high MI upon IL-23 stimulation (Fig. 3D + E).

In summary, the insertion of the known SOCS3 binding motifs from human or murine gp130 around Y476 in the IL-23R failed to induce strong SOCS3-negative feedback of IL-23 signal transduction. Instead, it underscores the intricacy of IL-23-induced signaling pathways and the selective role of diverse intracellular tyrosines.

### The gp130 intracellular domain renders IL-23 signaling sensitive for negative SOCS3 feedback

**Fig. 4:**
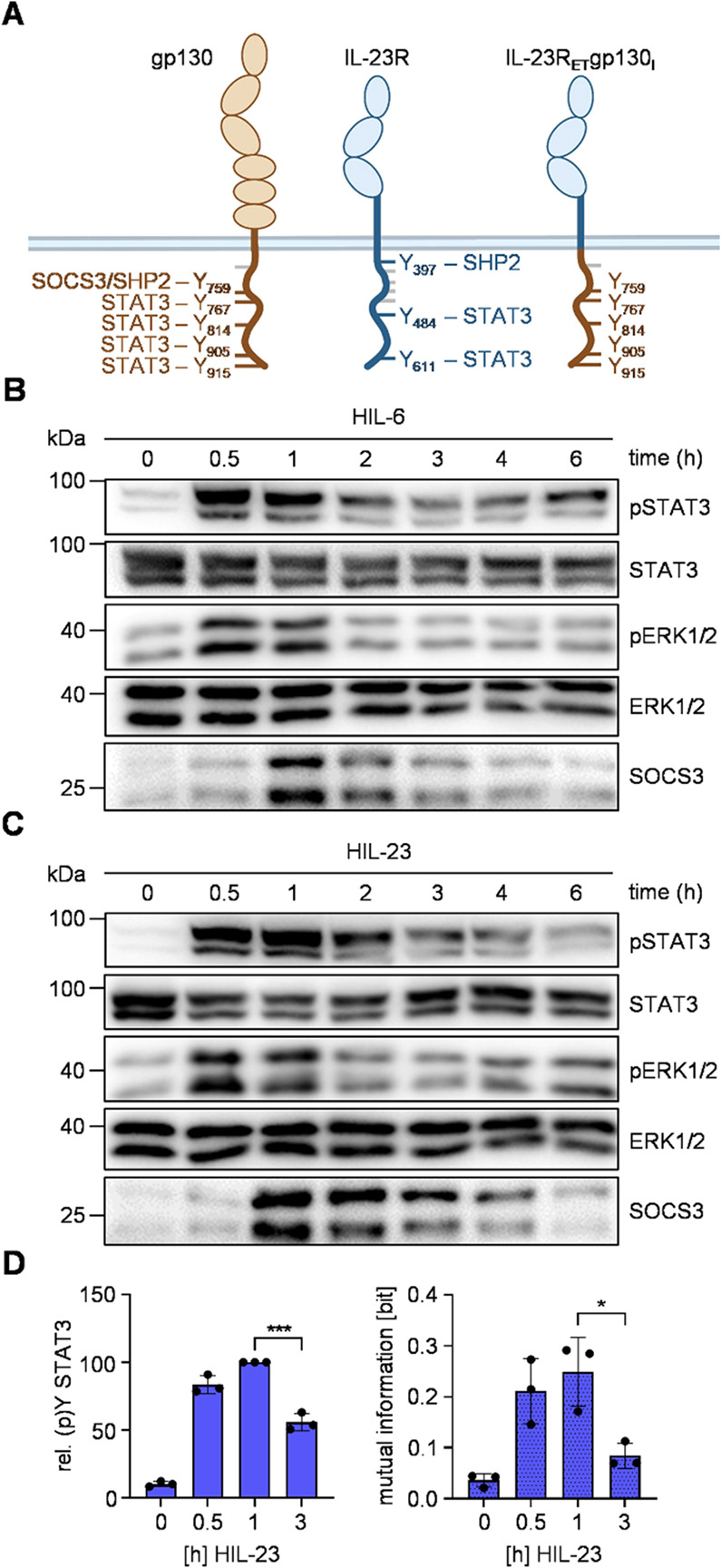
The IL-23R-gp130 chimera is sensitive for negative SOCS3 feedback.** A** Schematic illustration of gp130, IL-23R and IL-23R_ET_gp130_I_ chimera. Tyrosines implicated in signal transduction pathways are shown with respective signaling proteins. **B + C** STAT3 and ERK1/2 activation in Ba/F3-gp130 cells expressing IL-12Rβ1 and IL-23R_ET_gp130_I_ treated with HIL-6 (10 ng/ml) (**B**) or HIL-23 (10 ng/ml) (**C**) for indicated time points or left untreated. Equal amounts of proteins (50 µg/lane) were analyzed via specific antibodies detecting phospho-STAT3 and STAT3, phospho-ERK1/2 and ERK1/2, and SOCS3. Western blot data shows one representative experiment out of three. **D** Ba/F3-gp130-IL-12Rβ1-IL-23R_ET_gp130_I_ cells were stimulated with HIL-23 for the specified times. STAT3 expression (not shown) and STAT3-Y705 phosphorylation were assessed in parallel by multiplexed intracellular flow cytometry using fluorophore-coupled antibodies. For each independent time-point, the mean fluorescence intensity (MFI) of STAT3-Y705 staining was calculated from the single-cell data. The maximal MFI per experiment was normalized to 100%. Mutual information (MI) between STAT3 expression (not shown) and STAT3-Y705 phosphorylation was calculated from raw flow cytometry data in respective Ba/F3 cells stimulated with HIL-23 for the indicated times. Data represent mean ± SD from *n* = 3 experiments. Statistical analysis used one-way ANOVA, followed by Tukey correction, ***p ≤ 0.001, *p ≤ 0.05, ns not significant

Given the ineffectiveness of previous approaches to employ synthetic negative feedback by SOCS3 regulation of IL-23R, a broader approach was devised to demonstrate that the intracellular segment of a cytokine receptor is the sole mediator of negative SOCS3 regulation. Consequently, the complete extracellular (E) and transmembrane (T) domain of IL-23R was fused with the intracellular (I) domain of gp130, resulting in the formation of IL-23R_ET_gp130_I_. Cells that express the natural IL-12Rβ1 and the IL-23R_ET_gp130_I_ chimera should be activatable with IL-23 leading to intracellular IL-6 signaling. Conclusively, this IL-23-induced synthetic signaling should be subject to negative regulation by SOCS3. Thus, we generated Ba/F3-gp130 cells expressing IL-12Rβ1 and IL-23R_ET_gp130_I_ chimera to alter STAT3 and ERK1/2 activation kinetics upon HIL-23 administration (Fig. 4A [49], Additional Fig. 7). These cells have been stimulated with either HIL-6 or HIL-23 for up to 6 h and analyzed for activation of STAT3 and ERK1/2. Both cytokines induced rapid transient phosphorylation of STAT3 and ERK1/2 accompanied by expression of SOCS3 in later phases (Fig. 4B + C, Additional Fig. 6B). Additionally, replacing the intracellular part of IL-23R with gp130 restores robustness of late HIL-23-induced STAT3 activation, as evidenced by reduced MI over time (Fig. 4D). Thus, the sensitivity of the IL-23R to SOCS3 feedback solely depends on the intracellular part of the receptor and is not affected by extracellular and transmembrane domains.

**Fig. 5:**
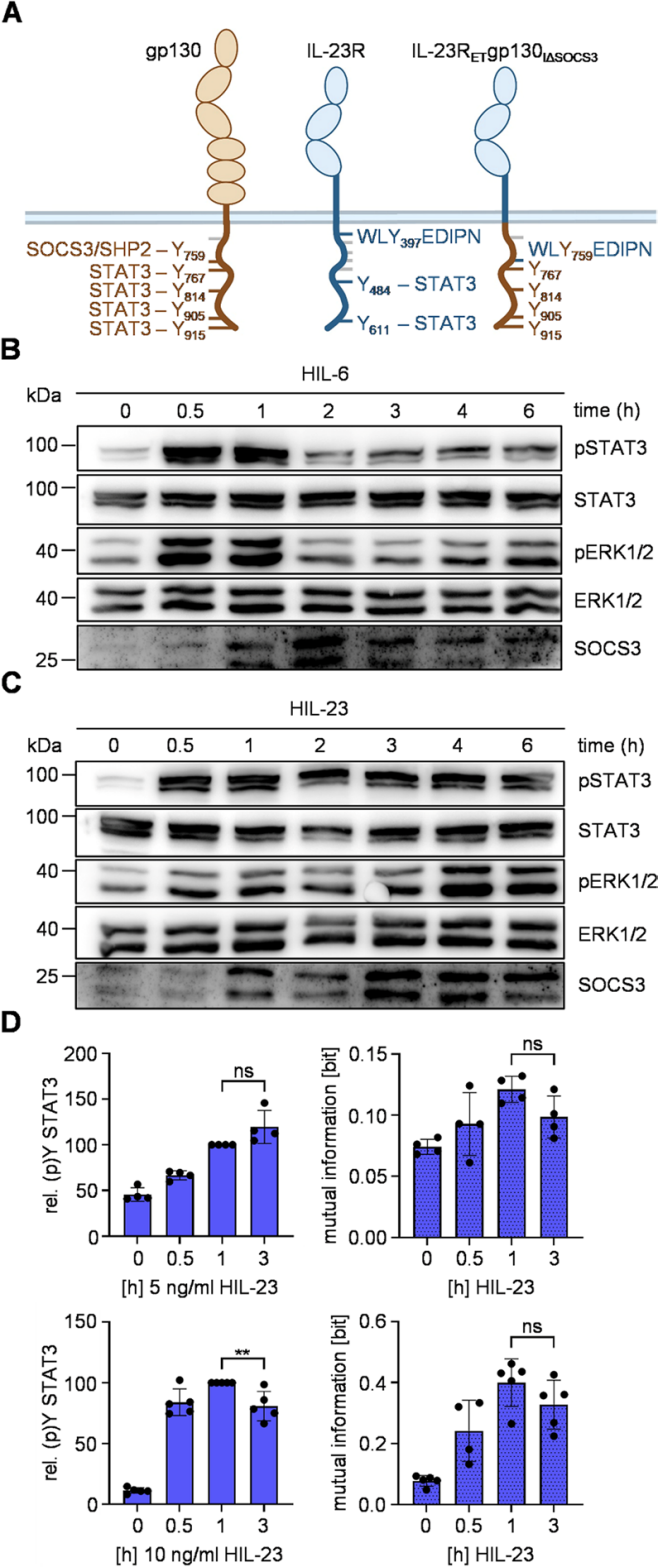
The IL-23R-gp130 chimera without SOCS3 binding motif is insensitive for negative SOCS3 feedback.** A** Schematic illustration of gp130, IL-23R and IL-23R_ET_gp130_IΔSOCS3_ chimera. Tyrosines implicated in signal transduction pathways are shown with respective signaling proteins. **B + C** STAT3 and ERK1/2 activation in Ba/F3-gp130 cells expressing IL-12Rβ1 and IL-23R_ET_gp130_IΔSOCS3_ treated with HIL-6 (10 ng/ml) (**B**) or HIL-23 (10 ng/ml) (**C**) for indicated time points or left untreated. Equal amounts of proteins (50 µg/lane) were analyzed via specific antibodies detecting phospho-STAT3 and STAT3, phospho-ERK1/2 and ERK1/2, and SOCS3. Western blot data shows one representative experiment out of three. **D** Ba/F3-gp130-IL-12Rβ1-IL-23R_ET_gp130_IΔSOCS3_ cells were stimulated with HIL-23 for the specified times. STAT3 expression (not shown) and STAT3-Y705 phosphorylation were assessed in parallel by multiplexed intracellular flow cytometry using fluorophore-coupled antibodies. For each independent time-point, the mean fluorescence intensity (MFI) of STAT3-Y705 staining was calculated from the single-cell data. The maximal MFI per experiment was normalized to 100%. Mutual information (MI) between STAT3 expression (not shown) and STAT3-Y705 phosphorylation was calculated from raw flow cytometry data in respective Ba/F3 cells stimulated with HIL-23 for the indicated times. Data represent mean ± SD from *n* = 4 (5 ng/ml) and *n* = 5 (10 ng/ml) experiments. Statistical analysis used one-way ANOVA, followed by Tukey correction, **p ≤ 0.01, ns not significant

Consequently, replacement of the gp130 SOCS3/SHP2 binding motif around Y759 within the IL-23R_ET_gp130_I_ chimera by the amino acids surrounding the SHP2 binding motif around Y397 of IL-23R (WLYEDIPN) named IL-23R_ET_gp130_IΔSOCS3_, should make this chimeric receptor again insensitive to SOCS3 feedback (Fig. 5A). Ba/F3-gp130 cells expressing IL-12Rβ1 and the IL-23R_ET_gp130_IΔSOCS3_ chimera have been generated (Additional Fig. 7) and were stimulated with either HIL-6 or HIL-23. Both HIL-6 and HIL-23 induce SOCS3 expression but only HIL-6-induced signaling was transient (Fig. 5B, Additional Fig. 6B). In contrast, HIL-23 stimulation of Ba/F3-gp130-IL-12Rβ1-IL-23R_ET_gp130_IΔSOCS3_ cells induced a sustained activation of STAT3 and ERK1/2 (Fig. 5C). This effect was supported by single-cell flow cytometry data. In contrast to signaling initiated by the IL-23R_ET_gp130_I_ chimera (Fig. 4), only a slight reduction of late STAT3 activation was detected 3 h after stimulation with high doses of HIL-23 (10 ng/ml). In line, MI was not significantly reduced over time (Fig. 5D). This highlights the importance of the VQYSTVVH motif in the intracellular part of gp130 for negative feedback and robustness mediated by SOCS3. Notably, replacement of the SOCS3/SHP2 binding motif in gp130 by the SHP2-only binding motif of IL-23R uncouples gp130-dependent ERK-activation from negative feedback by SOCS3 (Fig. 5C).

**Fig. 6:**
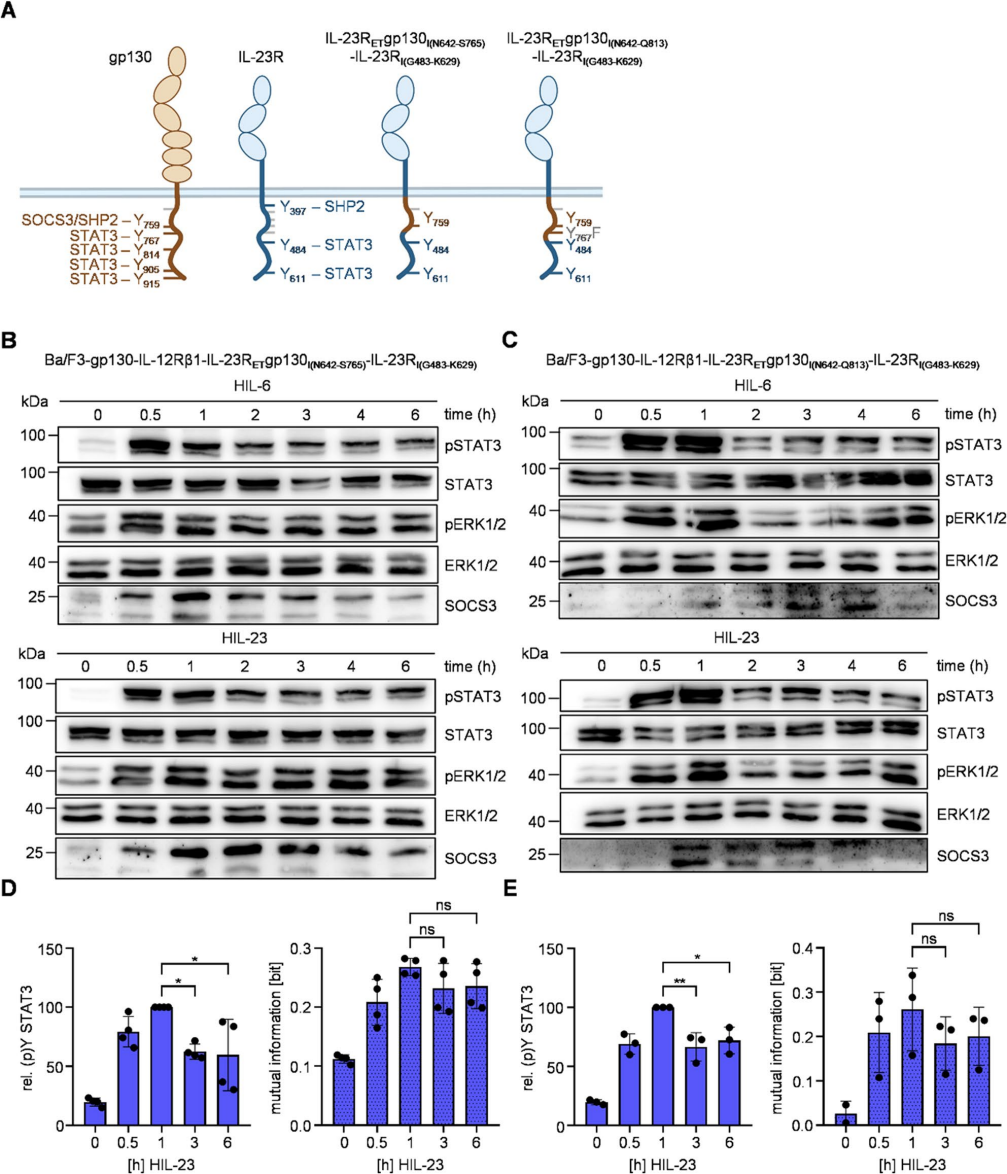
Membrane proximal intracellular regions of gp130 are important for negative SOCS3 feedback.** A** Schematic illustration of gp130, IL-23R and modified variants with membrane proximal regions of gp130. Tyrosines implicated in signal transduction pathways are shown with respective signaling proteins. **B + C** STAT3 and ERK1/2 activation in Ba/F3-gp130 cells expressing IL-12Rβ1 and IL-23R_ET_gp130_I(N642−S765)_-IL-23R_I(G483−K629)_ (**B**) or IL-23R_ET_gp130_I(N642−Q813)_-IL-23R_I(G483−K629)_ (**C**) treated with HIL-6 (10 ng/ml) or HIL-23 (10 ng/ml) for indicated time points or left untreated. Equal amounts of proteins (50 µg/lane) were analyzed via specific antibodies detecting phospho-STAT3 and STAT3, phospho-ERK1/2 and ERK1/2, and SOCS3. Western blot data shows one representative experiment out of three. **D** + **E** Ba/F3-gp130-IL-12Rβ1-IL-23R_ET_gp130_I(N642−S765)_-IL-23R_I(G483−K629)_ (**D**) or Ba/F3-gp130-IL-12Rβ1-IL-23R_ET_gp130_I(N642−Q813)_-IL-23R_I(G483−K629)_ (**E**) cells were stimulated with HIL-23 for the specified times. STAT3 expression (not shown) and STAT3-Y705 phosphorylation were assessed in parallel by multiplexed intracellular flow cytometry using fluorophore-coupled antibodies. For each independent time-point, the mean fluorescence intensity (MFI) of STAT3-Y705 staining was calculated from the single-cell data. The maximal MFI per experiment was normalized to 100%. Mutual information (MI) between STAT3 expression (not shown) and STAT3-Y705 phosphorylation was calculated from raw flow cytometry data in respective Ba/F3 cells stimulated with HIL-23 for the indicated times. Data represent mean ± SD from *n* = 4 (**D**) and *n* = 3 (**E**) experiments. Statistical analysis used one-way ANOVA, followed by Tukey correction, **p ≤ 0.01, *p ≤ 0.05, ns not significant

Next, we gradually reduced the proportion of gp130 in the synthetic IL-23R_ET_gp130_I_ chimera to define the minimum requirements for negative SOCS3 regulation. These IL-23R variants include the intracellular most C-terminal part of IL-23R from G483 to K629 containing the critical signal transducing tyrosines Y484 and Y611. The membrane proximal intracellular regions were however derived from the gp130 cytosolic domain (N642-S765 or N642-Q813) resulting in the chimeric receptors IL-23R_ET_gp130_I(N642−S765)_-IL-23R_I(G483−K629)_ and IL-23R_ET_gp130_I(N642−Q813)_-IL-23R_I(G483−K629)_. In the latter variant, Y767 of gp130 has been mutated to phenylalanine to prevent gp130-mediated STAT3 phosphorylation. This allows only Y484 and Y611 within IL-23R, to be used for STAT3 activation (Fig. 6A). The respective Ba/F3-gp130 cells have been generated and expression of IL-12Rβ1 and the IL-23R chimeras was confirmed by flow cytometry (Additional Fig. 7). Cells were stimulated with HIL-6 or HIL-23 and intracellular signaling was analyzed. As expected, HIL-6-induced STAT3 and ERK1/2 activation were transient. Treatment of the cells with HIL-23 also resulted in induction of SOCS3 expression and thus transient STAT3 phosphorylation (Fig. 6B + C). This negative feedback inhibition was also demonstrated by intracellular staining of phosphorylated STAT3. In line, MI at late time points was slightly, however not significantly, reduced compared to early time points of stimulation with HIL-23. This shows, that inclusion of membrane proximal intracellular regions of gp130 into IL-23R has the potential to increase robustness of IL-23-induced STAT3 phosphorylation at late time points but that differences between signaling induced by gp130 and IL-23R exist. (Fig. 6D + E). Interestingly, the ERK1/2 activation pattern varied between the two receptor variants. Cells expressing IL-23R_ET_gp130_I(N642−Q813)_-IL-23R_I(G483−K629)_ demonstrated a comparable transient ERK1/2 phosphorylation pattern following HIL-23 and HIL-6 treatment, while HIL-23-induced ERK1/2 activation was sustained in cells expressing IL-23R_ET_gp130_I(N642−S765)_-IL-23R_I(G483−K629)_.

In summary, we show that the engineering of synthetic IL-23 receptors that render IL-23 signaling responsive to negative feedback by SOCS3 also ensured robustness of STAT3 activation and provided valuable insights into the intricate mechanisms of IL-23 signal transduction.

## Discussion

IL-23 has been identified as a pivotal cytokine in the expansion and survival of TH17 cells. Nonpathogenic TH17 cells have been demonstrated to modulate barrier functions and regulate microbial invasion at mucosal surfaces. Conversely, pathogenic TH17 cells have been shown to induce tissue inflammation and autoimmunity [50]. The expression of the IL-23R serves as a distinguishing factor between pathogenic and nonpathogenic type 17 cells. Pathogenic cells exhibit elevated levels of IL-23R expression [51]. Accordingly, IL-23 is the dominant regulatory cytokine in a cluster of immune-mediated inflammatory diseases, but due to its pleiotropic nature it also plays an important role in other diseases [50]. Various options for targeted therapy of IL-23 signal transduction are being pursued [52]. Novel gene-based methodologies for the selective modulation of IL-23 signal transduction offer a compelling avenue for further exploration. However, to develop these approaches the understanding of IL-23 signaling is essential. Negative feedback regulation of IL-23 signaling via SOCS3 is controversially discussed [26, 53]. We demonstrated that SOCS3 does not influence IL-23-induced STAT3 activation by murine and synthetic receptors [21, 22]. In the current study we extend this result to human IL-23R signaling and support our previous finding by additional analysis of ERK phosphorylation and by information theoretic measures. Recently, it was shown that robustness of IL-6-induced STAT3 activation is achieved by different mechanisms including repression of basal STAT3 activation by SHP2 [14, 15] and feedback inhibition by SOCS3 [29]. Billing et al. demonstrated that SOCS3, and the phosphorylation of STAT3 at serine 727 facilitated robust activation of STAT3 by constraining tyrosine phosphorylation of STAT3. Furthermore, the robustness of the system was ensured in the presence of low levels of cytokine and high levels of STAT3 expression. These mechanisms complemented each other at varying time scales and cytokine doses [29]. It is so far unknown, whether robustness of STAT3 activation induced by other natural IL-6-type cytokines or even engineered cytokines is also achieved by comparable mechanisms. Here, we show that robustness of natural HIL-23-induced STAT3 activation against heterogeneity in STAT3 follows a different kinetics than HIL-6-induces STAT3 activation. While HIL-6 only transiently reduced robustness of STAT3 activation (Fig. 1D [29]), robustness of HIL-23-induced STAT3 is also reduced at late time-points (Fig. 1E). Notably, rendering IL-23R sensitive to SOCS3 feedback also increased robustness of late HIL-23-induced STAT3 activation against heterogenous STAT3 expression (Figs. 4D and 6D + E). This shows, that signaling induced by engineered cytokine receptors quantitively and qualitatively differs from signaling induced by natural cytokines. Thus, differences in robustness are most likely a general feature among the novel characteristics and functions of engineered cytokine receptors. These properties will affect the success of engineered cytokine receptors e.g., in therapeutic approaches and could serve as new target variables in the development of engineered cytokines.

Constitutive and prolonged phosphorylation of STAT3 has been observed to be associated with cancerous and chronic inflammatory diseases. Consequently, the targeted induction of SOCS3-mediated STAT3 suppression has emerged as a novel approach to limit the development of pathogenic TH17 cells. Nowadays, synthetic cytokine receptors have become indispensable in terms of novel therapeutic options. Such receptors are engineered to transfer information within and between cells by modifying either the sensing domains of native receptors with other ligand-binding domains, or the transmembrane and intracellular domains [54, 55]. Chimeric receptors expressing the ECD of one protein and the ICD of another have long been used to isolate the effects of ligand binding from signaling [56]. We already used this system to engineer cytokine receptors that were responsive to extracellular IL-12/IL-23 stimulation and induced intracellular IL-35/IL-39-signal transduction [49]. In the present study, we generated chimeric IL-23R that were responsive to extracellular IL-23 stimulation and induced intracellular gp130 signaling. These receptors induced rapid transient phosphorylation of STAT3 and ERK1/2 upon IL-23 stimulation accompanied by expression of SOCS3. The length of the inserted gp130 segment within the IL-23R ICD appears to play a role in the dynamics of signal transduction. Additionally, we confirmed the importance of the VQYSTVHH motif within human gp130 for SOCS3 induced feedback mechanism through exchange to WLYEDIPN in the IL-23R_ET_gp130_IΔSOCS3_ variant (Fig. 5B). Earlier data showed that peptides centered around murine gp130’s pY757 (STASTVEpYSTVVHSG) or human pY759 (TSSTVQpYSTVVHSG) could efficiently capture SOCS3 in a biotin-streptavidin pull-down assay [43, 44]. However, simple substitution of the WLYEDIPN motif in human IL-23R, surrounding the Y397, with the gp130 SOCS3 binding motif did not automatically lead to transient activation of STAT3. Interestingly, cells expressing this motif around Y397 in a modified IL-23R were unresponsive to IL-23 (Fig. 2B). This is probably due to the lack of activation of JAK2 and subsequently lack of phosphorylation of STAT3 and SHP2. The specific exchange of amino acids within the WLYEDIPN motif supports that this amino acid sequence is crucial for the activity of the IL-23R associated Janus kinase JAK2 (Fig. 2H). We already showed that association of JAK2 with the IL-23R is not mediated via classical Box1/Box2 motifs. Additionally, mutation of membrane-proximal R400 (human: R381) and Y416 (human: Y397) in murine IL-23R reduced activation of JAK2 and STAT3, respectively [46]. Furthermore, our previous studies have demonstrated that Y416 in murine and Y397 in human IL-23R, are involved in the activation of PI3K/Akt and the MAPK pathway leading to STAT3-independent proliferation of Ba/F3 cells upon stimulation with IL-23 [21]. These results corroborate the conclusions of our present study, which demonstrated that WLYEDIPN is an indispensable motif for IL-23R signal transduction.

The crystal structure of a ternary complex consisting of SOCS3, the kinase domain of JAK2, and a fragment of gp130 was determined by Kershaw and colleagues. They showed that SOCS3 simultaneously binds to JAK2 and gp130 using two different interfaces. While the phospho-tyrosine-binding groove of the SOCS3 SH2 domain is occupied by the receptor, JAK2 binds to a noncanonical surface within SOCS3 in a phospho-tyrosine-independent manner [11]. Gp130 has a single binding site for SOCS3 centered around pY757 (murine) or pY759 (human) [44]. Our studies clearly show that the amino acids surrounding Y759 in gp130 are essential for the ability of SOCS3 to repress gp130-mediated signaling (Fig. 5B). Interaction between of SOCS3 and JAK2 is mediated through the GQM motif (Gly1071, Gln1072 and Met1071) within the JAK insertion loop, which is necessary for inhibition of JAKs by SOCS3 [57]. SOCS3 binds with its SH2 domain, the extended SH2 subdomain and the KIR to the GQM motif [11]. The membrane-proximal Box1 motif of gp130 (PNVPDP) is required but not sufficient for interaction with JAK2 [58]. The SOCS3 binding motif of gp130, which surrounds pY759, is located C-terminal to the box motifs. In contrast, even though the involvement of JAK2 in IL-23 signaling is widely accepted, classical Box motifs are not described within the IL-23 receptor, indicating the presence of another interaction side between IL-23R and JAK2. This different composition of the intracellular parts of gp130 and IL-23 might explain, why SOCS3 does not inhibit IL-23-induced JAK2 activity.

As early as the 1990 s, it was demonstrated using chimeric receptors that tyrosine residues in the C-terminal region of gp130 are important for the proliferation of Ba/F3 cells, as well as for growth arrest and macrophage differentiation in a myeloid leukemia cell line [59, 60]. Substitution of Y759 to phenylalanine in an EpoR-gp130 chimera revealed that the lack of SHP2 activation leads to enhanced SOCS3 expression, and the efficient inhibition of STAT3 activation by SOCS3 requires Y759 in gp130 [43]. Ba/F3 cells expressing gp130^Y759F^ showed an enhanced STAT activation compared to cells with wild-type human gp130 [61]. Additionally, these cells were much more responsive to low doses of IL-6 and expressed more SOCS3 mRNA. Mice carrying the corresponding gp130 Y757F mutation showed impaired IL-6-dependent activation of the SHP2-Ras-ERK pathway, but not of the STAT1/3 pathway [62]. Instead, STAT3 phosphorylation was enhanced in the liver and stomach of IL-6- or IL-11-treated gp130^Y757F^ mice. Furthermore, STAT3 hyperactivation promotes gastric cancer in gp130 Y757F mutant mice [63]. Conversely, the disruption of the SOCS3 binding site of the IL-23R-gp130 chimera in our analyses resulted in a receptor variant with sustained STAT3 and ERK phosphorylation (Fig. 5B + C). By this, we for the first time generated a gp130 variant that does not exhibit negative STAT3 feedback, but rather activates the MAPK pathway.

## Conclusions

Engineering of cytokine receptors and analysis of intracellular signaling pathways by information theoretic measures are important tools for rationale cytokine/cytokine-receptor design. Fine-tuning the signal transduction of cytokine receptors, illustrated here using the induction of a negative feedback mechanism implied by a chimeric IL-23R, is a critical prerequisite for the utilization of cytokine receptors in novel therapeutic modalities. In this study, we demonstrated for the first time using the example of IL-23R that a SOCS3-insensitive receptor becomes sensitive through engineering of the intracellular domain. Conversely, the ability of SOCS3 to regulate the IL-6 transducing receptor gp130 was found to be abolished by altering the SOCS3 binding site. In the future, it will be interesting to see to what extent chimeric receptors with modified signal transduction will be used specifically in gene therapies.

## Supplementary Information

Additional figure 1. Analysis of Ba/F3-gp130-IL-12Rβ1-IL-23R cells with regard to receptor and SOCS3 expression. A) Flow cytometry analysis of IL-23 receptors on the surface of Ba/F3-gp130-IL-12Rβ1-IL-23R cells, indicated as solid line. Expression was detected via antibodies against extracellular domains of IL-12Rβ1 (left panel) or IL-23R (right panel). Gray-shade area indicates non-transfected Ba/F3-gp130 cells (negative control). B) Quantification of SOCS3 mRNA expression in stimulated Ba/F3-gp130-IL-12Rβ1-IL-23R cells. The specified times were used for stimulation with HIL-6 (10 ng/ml) or HIL-23 (10 ng/ml). ***p ≤ 0.001, ns not significant. Additional figure 2. SOCS3 interacts with IL-23R. A) Co-IP of FLAG-tagged SOCS3 and full-length IL-23R using ANTI-FLAG® M2 affinity gel. One of two independent experiments is shown. L, lysates; IP, co-immunoprecipitates. B) Co-IP of FLAG-tagged SOCS3 and IL-23R deletion variants using ANTI-FLAG® M2 affinity gel. One of two independent experiments is shown. L, lysates; IP, co-immunoprecipitates. Additional figure 3. IL-23 receptor surface expression. Flow cytometry analysis of IL-23 receptors on the surface of Ba/F3-gp130 cells, indicated as solid line. Expression was detected via antibodies against extracellular domains of IL-12Rβ1 (left panel) or IL-23R (right panel). Gray-shade area indicates non-transfected Ba/F3-gp130 cells (negative control). Additional figure 4. SOCS3 induced negative feedback of Ba/F3-gp130 cell lines stimulated with HIL-6. STAT3 and ERK1/2 activation in Ba/F3-gp130 cells expressing IL-12Rβ1 and IL-23RW395V (A), IL-23RI400V-P401V (B) or IL-23RW395V-I400V-P401V (C) treated with HIL-6 (10 ng/ml) for indicated time points or left untreated. Equal amounts of proteins (50 μg/lane) were analyzed via speciﬁc antibodies detecting phospho-STAT3 and STAT3, phospho-ERK1/2 and ERK1/2, and SOCS3. Western blot data shows one representative experiment out of three. Additional figure 5. IL-23 receptor surface expression. Flow cytometry analysis of IL-23 receptors on the surface of Ba/F3-gp130 cells, indicated as solid line. Expression was detected via antibodies against extracellular domains of IL-12Rβ1 (left panel) or IL-23R (right panel). Gray-shade area indicates non-transfected Ba/F3-gp130 cells (negative control). Additional figure 6. Analysis of SOCS3 expression in stimulated Ba/F3-gp130-IL-12Rβ1-IL-23R cells. Quantification of SOCS3 mRNA expression in stimulated Ba/F3-gp130-IL-12Rβ1-IL-23R cells. The specified times were used for stimulation with HIL-6 (10 ng/ml) or HIL-23 (10 ng/ml). **p ≤ 0.01, **p ≤ 0.05, ***p ≤ 0.001, ns not significant. Additional figure 7. IL-23 receptor surface expression. Flow cytometry analysis of IL-23 receptors on the surface of Ba/F3-gp130 cells, indicated as solid line. Expression was detected via antibodies against extracellular domains of IL-12Rβ1 (left panel) or IL-23R (right panel). Gray-shade area indicates non-transfected Ba/F3-gp130 cells (negative control).

Supplementary Material 2

## Data Availability

All data generated or analyzed during the study are included in this published article [and its supplementary information files].

## Abbreviations

Akt: Protein kinase B
CIS: Cytokine-inducible Src homology 2 containing protein
ECD: Extracellular domain
ERK: Extracellular signal regulated kinase
GM-CSF: Granulocyte-macrophage colony-stimulating factor
gp130: Glycoprotein 130
ICD: Intracellular domain
IL-6: Interleukin 6
IL-12Rβ1: Interleukin 12 receptor β 1
IL-17: Interleukin 17
IL-22: Interleukin 22
IL-23: Interleukin 23
IL-23R: Interleukin 23 receptor
JAK: Janus kinase
KIR: Kinase inhibitory region
MAPK: Mitogen-activated protein kinase
NFκB: Nuclear factor kappa B
PIAS: Protein inhibitor of activated STAT
PTP: Protein tyrosine phosphatase
PI3K: Phosphoinositide 3-kinase
SH2: Src homology 2
SHP2: SH2-domain containing protein tyrosine phosphatase 2
SOCS: Suppressor of cytokine signaling
STAT: Signal transducer and activator of transcription
TGF: Transforming growth factor
TH17: T helper 17 cell
TNF: Tumor necrosis factor
MI: Mutual information
