# Supplementary material for: Receptor engineering constitutes feedback control and robustness of IL-23R signaling and highlights importance of intracellular cytokine receptor signaling motifs
Source: Cell Commun Signal. 2026 Jan 6;24:51. doi: 10.1186/s12964-025-02593-2 (PMC12849345; doi:10.1186/s12964-025-02593-2)
Supplement: Supplementary file 1 — Additional figure 1. Analysis of Ba/F3-gp130-IL-12Rβ1-IL-23R cells with regard to receptor and SOCS3 expression. A) Flow cytometry analysis of IL-23 receptors on the surface of Ba/F3-gp130-IL-12Rβ1-IL-23R cells, indicated as solid line. Expression was detected via antibodies against extracellular domains of IL-12Rβ1 (left panel) or IL-23R (right panel). Gray-shade area indicates non-transfected Ba/F3-gp130 cells (negative control). B) Quantification of SOCS3 mRNA expression in stimulated Ba/F3-gp130-IL-12Rβ1-IL-23R cells. The specified times were used for stimulation with HIL-6 (10 ng/ml) or HIL-23 (10 ng/ml). ***p ≤ 0.001, ns not significant. Additional figure 2. SOCS3 interacts with IL-23R. A) Co-IP of FLAG-tagged SOCS3 and full-length IL-23R using ANTI-FLAG® M2 affinity gel. One of two independent experiments is shown. L, lysates; IP, co-immunoprecipitates. B) Co-IP of FLAG-tagged SOCS3 and IL-23R deletion variants using ANTI-FLAG® M2 affinity gel. One of two independent experiments is shown. L, lysates; IP, co-immunoprecipitates. Additional figure 3. IL-23 receptor surface expression. Flow cytometry analysis of IL-23 receptors on the surface of Ba/F3-gp130 cells, indicated as solid line. Expression was detected via antibodies against extracellular domains of IL-12Rβ1 (left panel) or IL-23R (right panel). Gray-shade area indicates non-transfected Ba/F3-gp130 cells (negative control). Additional figure 4. SOCS3 induced negative feedback of Ba/F3-gp130 cell lines stimulated with HIL-6. STAT3 and ERK1/2 activation in Ba/F3-gp130 cells expressing IL-12Rβ1 and IL-23RW395V (A), IL-23RI400V-P401V (B) or IL-23RW395V-I400V-P401V (C) treated with HIL-6 (10 ng/ml) for indicated time points or left untreated. Equal amounts of proteins (50 μg/lane) were analyzed via speciﬁc antibodies detecting phospho-STAT3 and STAT3, phospho-ERK1/2 and ERK1/2, and SOCS3. Western blot data shows one representative experiment out of three. Additional figure 5. IL-23 receptor surface expressio [file 12964_2025_2593_MOESM1_ESM.docx]

**Additional files**

**
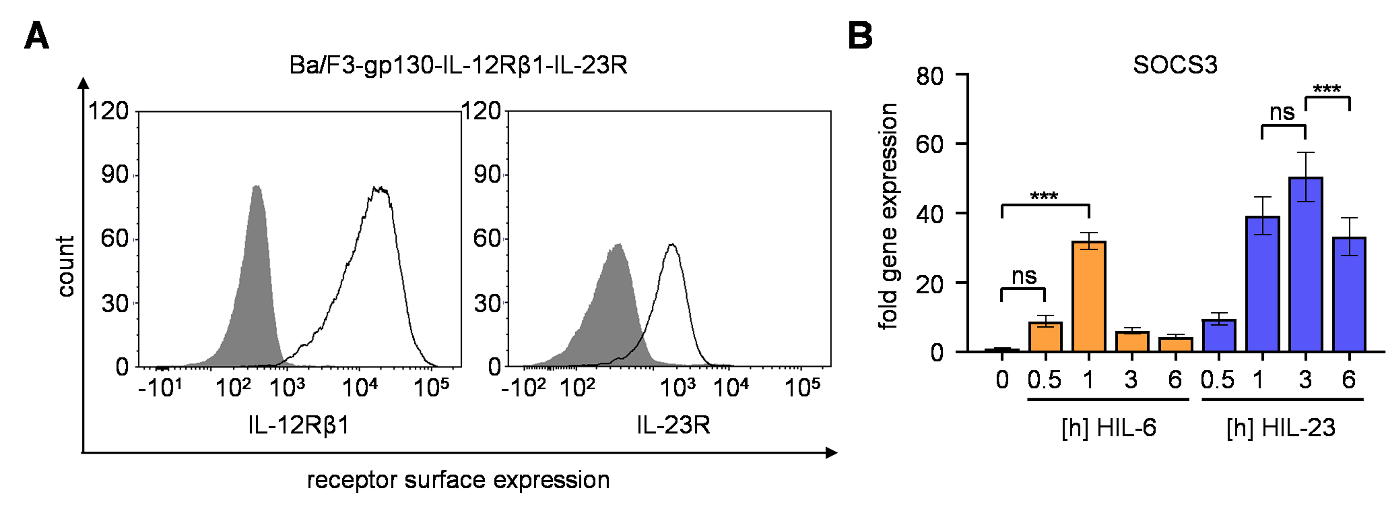
**

**Additional figure 1: Analysis of Ba/F3-gp130-IL-12Rβ1-IL-23R cells with regard to receptor and SOCS3 expression. A)** Flow cytometry analysis of IL-23 receptors on the surface of Ba/F3-gp130-IL-12Rβ1-IL-23R cells, indicated as solid line. Expression was detected via antibodies against extracellular domains of IL-12Rβ1 (left panel) or IL-23R (right panel). Gray-shade area indicates non-transfected Ba/F3-gp130 cells (negative control). **B)** Quantification of SOCS3 mRNA expression in stimulated Ba/F3-gp130-IL-12Rβ1-IL-23R cells. The specified times were used for stimulation with HIL-6 (10 ng/ml) or HIL-23 (10 ng/ml). ***p ≤ 0.001, ns not significant.


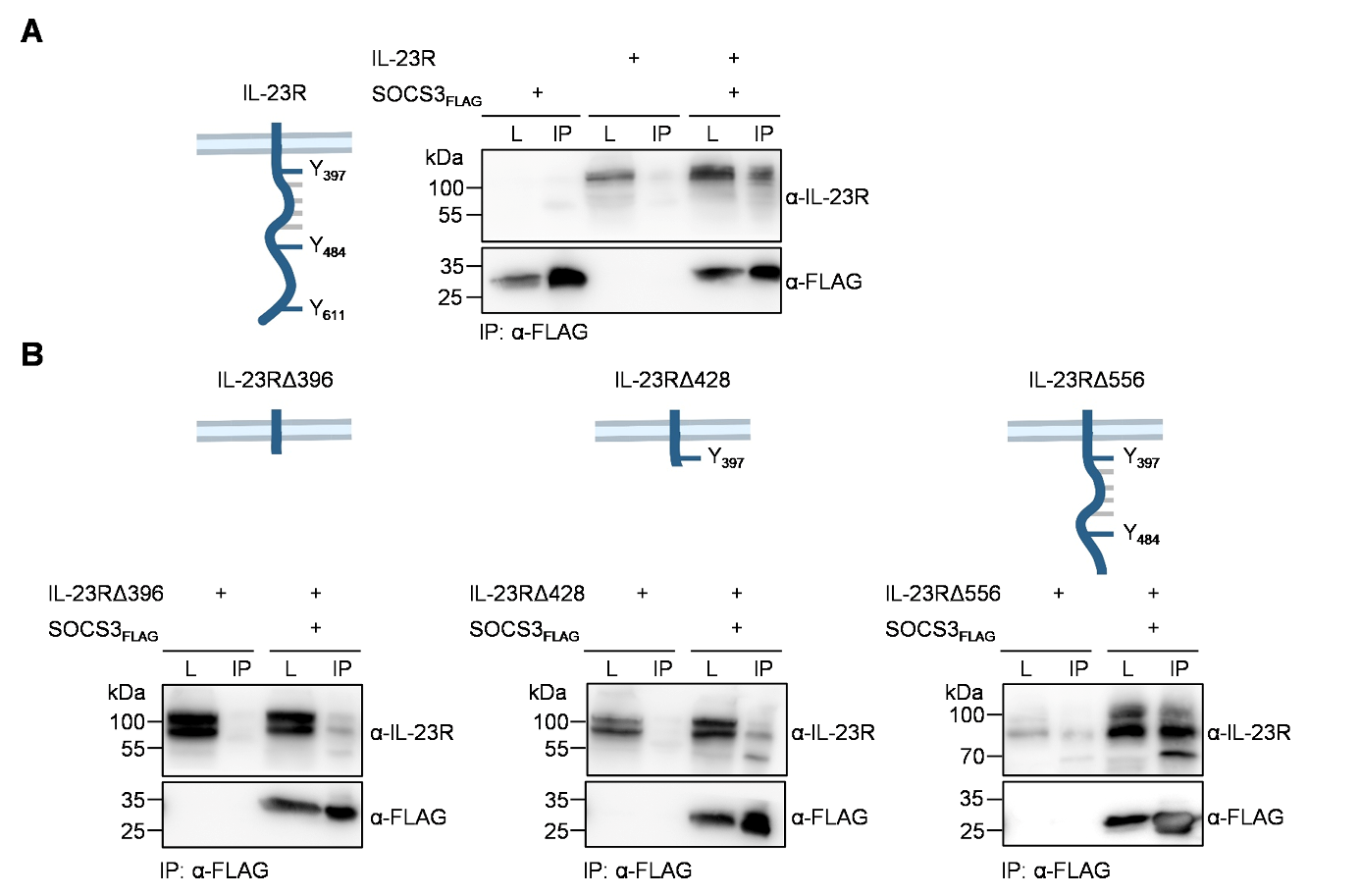


**Additional figure 2: SOCS3 interacts with IL-23R. A)** Co-IP of FLAG-tagged SOCS3 and full-length IL-23R using ANTI-FLAG^®^ M2 affinity gel. One of two independent experiments is shown. L, lysates; IP, co-immunoprecipitates. **B)** Co-IP of FLAG-tagged SOCS3 and IL-23R deletion variants using ANTI-FLAG^®^ M2 affinity gel. One of two independent experiments is shown. L, lysates; IP, co-immunoprecipitates.


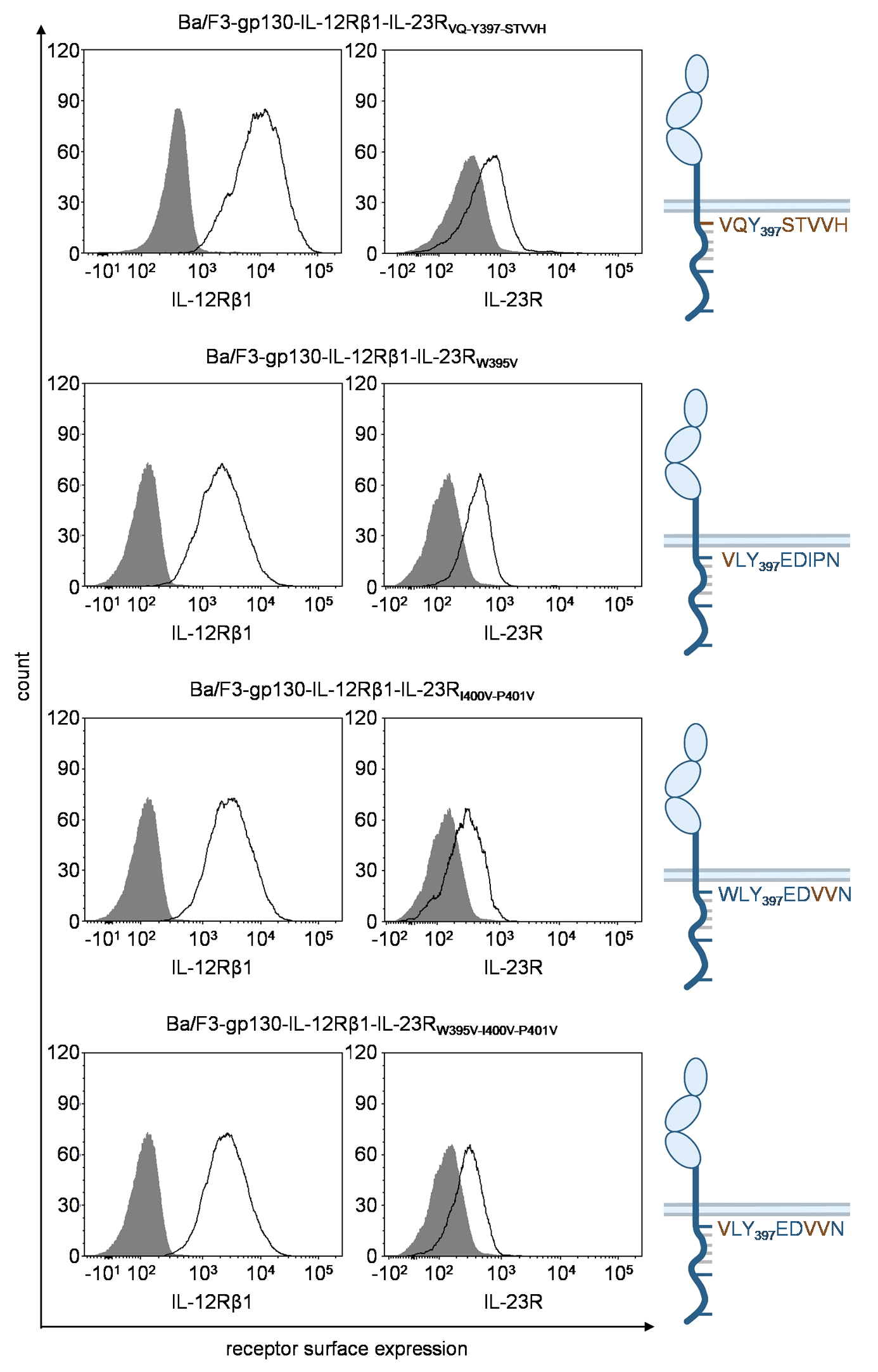


**Additional figure 3: IL-23 receptor surface expression.** Flow cytometry analysis of IL-23 receptors on the surface of Ba/F3-gp130 cells, indicated as solid line. Expression was detected via antibodies against extracellular domains of IL-12Rβ1 (left panel) or IL-23R (right panel). Gray-shade area indicates non-transfected Ba/F3-gp130 cells (negative control).


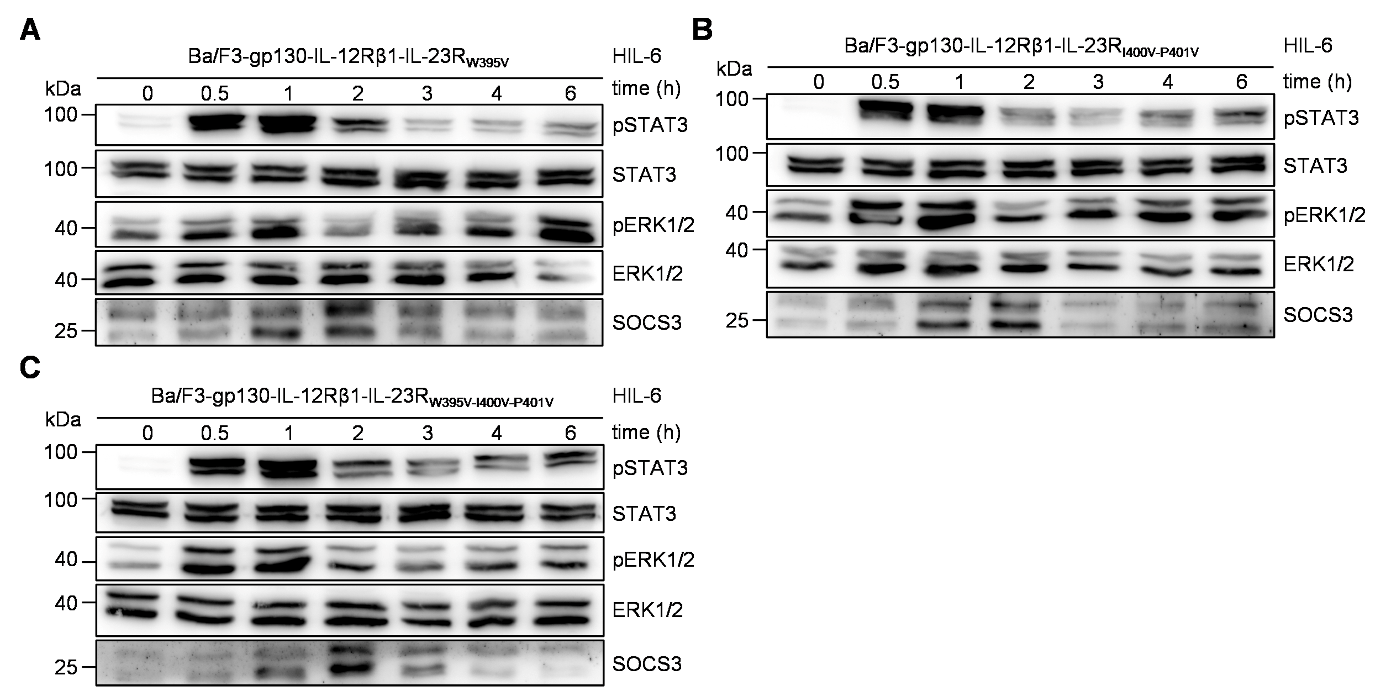


**Additional figure 4: SOCS3 induced negative feedback of Ba/F3-gp130 cell lines stimulated with HIL-6.** STAT3 and ERK1/2 activation in Ba/F3-gp130 cells expressing IL-12Rβ1 and IL-23R_W395V_ (A), IL-23R_I400V-P401V_ (B) or IL-23R_I400V-P401W395V_ (C) treated with HIL-6 (10 ng/ml) for indicated time points or left untreated. Equal amounts of proteins (50 μg/lane) were analyzed via speciﬁc antibodies detecting phospho-STAT3 and STAT3, phospho-ERK1/2 and ERK1/2, and SOCS3. Western blot data shows one representative experiment out of three.


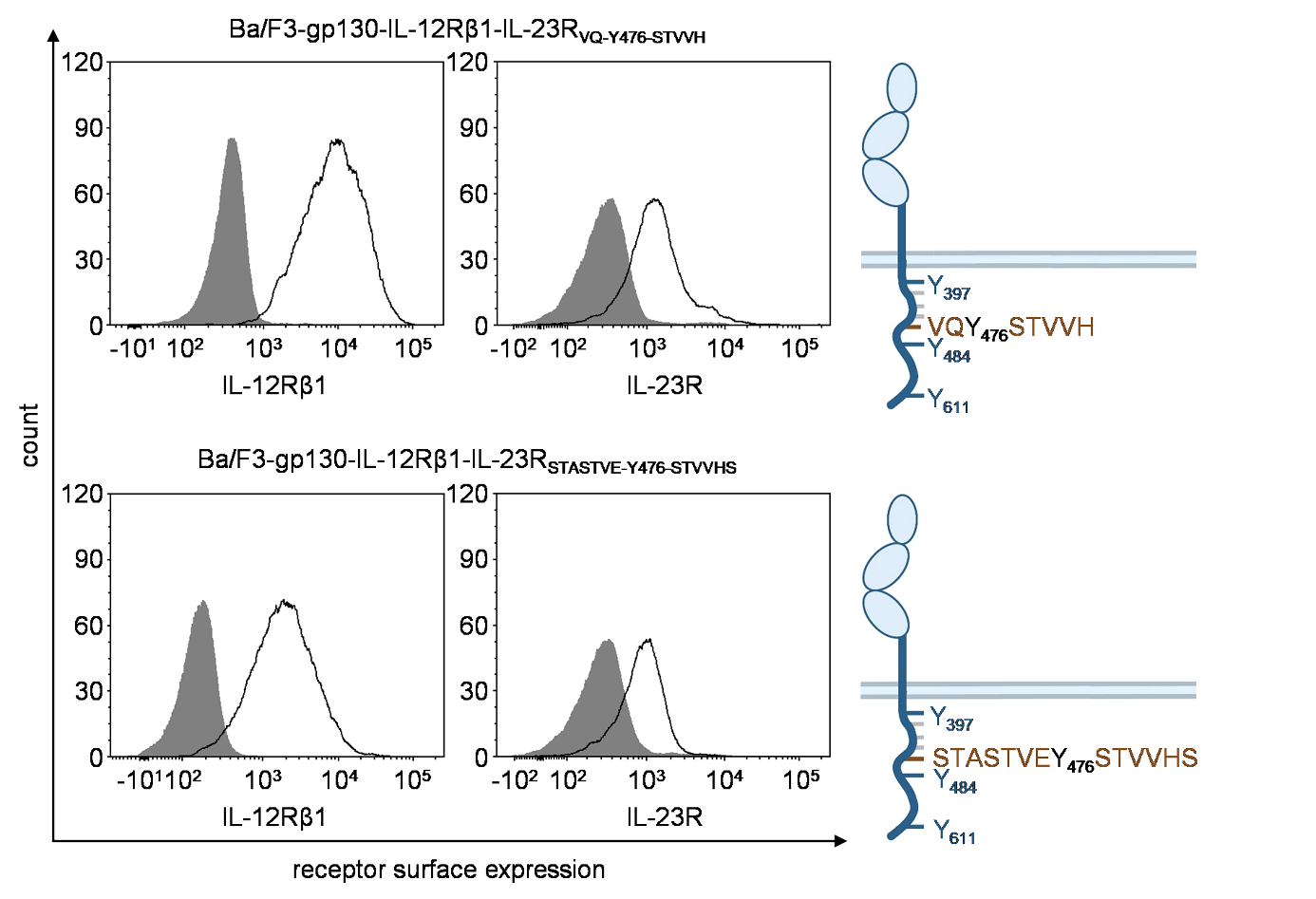


**Additional figure 5: IL-23 receptor surface expression.** Flow cytometry analysis of IL-23 receptors on the surface of Ba/F3-gp130 cells, indicated as solid line. Expression was detected via antibodies against extracellular domains of IL-12Rβ1 (left panel) or IL-23R (right panel). Gray-shade area indicates non-transfected Ba/F3-gp130 cells (negative control).


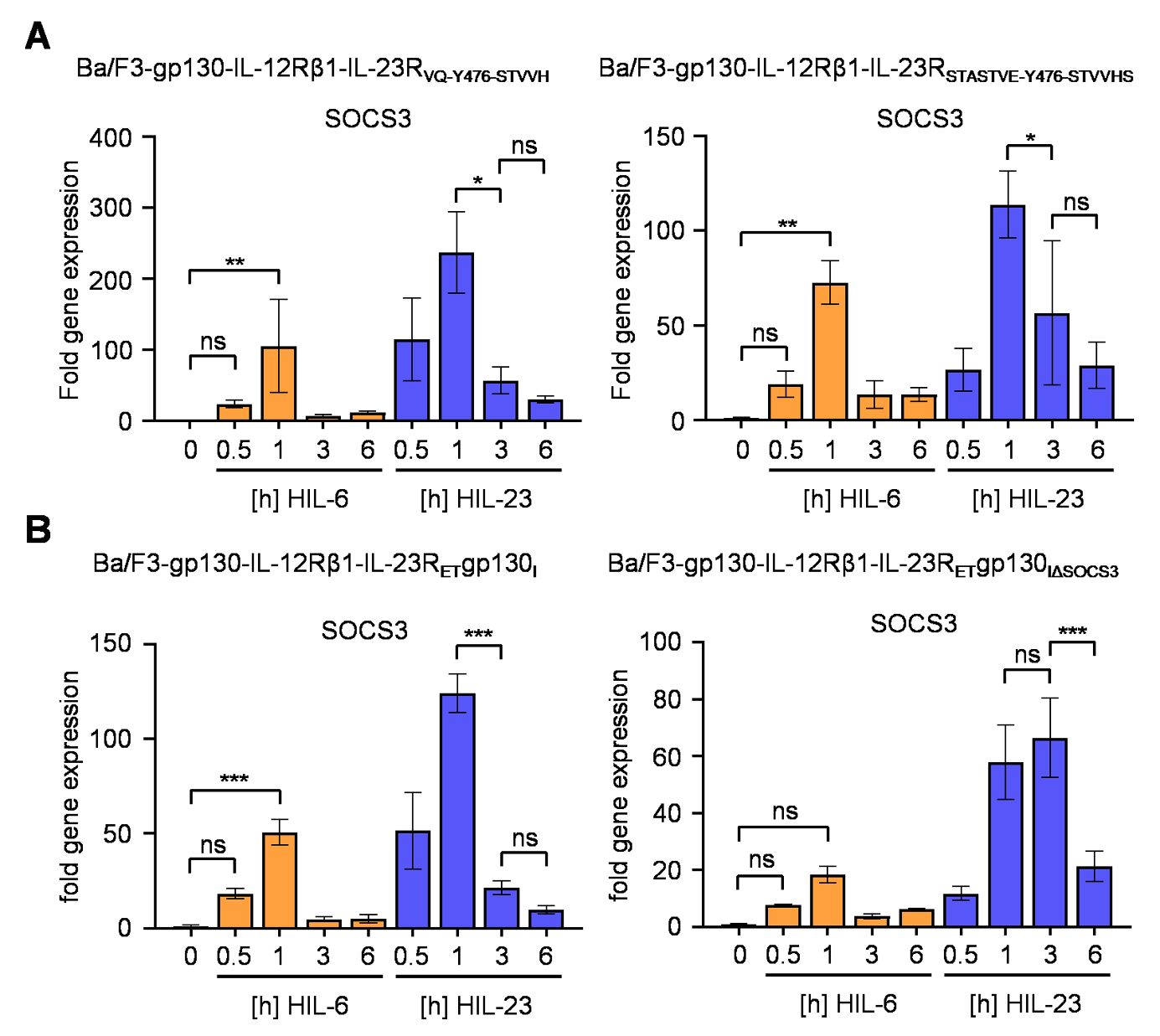


**Additional figure 6: Analysis of SOCS3 expression in stimulated Ba/F3-gp130-IL-12Rβ1-IL-23R cells.** Quantification of SOCS3 mRNA expression in stimulated Ba/F3-gp130-IL-12Rβ1-IL-23R cells. The specified times were used for stimulation with HIL-6 (10 ng/ml) or HIL-23 (10 ng/ml). **p ≤ 0.01, **p ≤ 0.05, ***p ≤ 0.001, ns not significant.


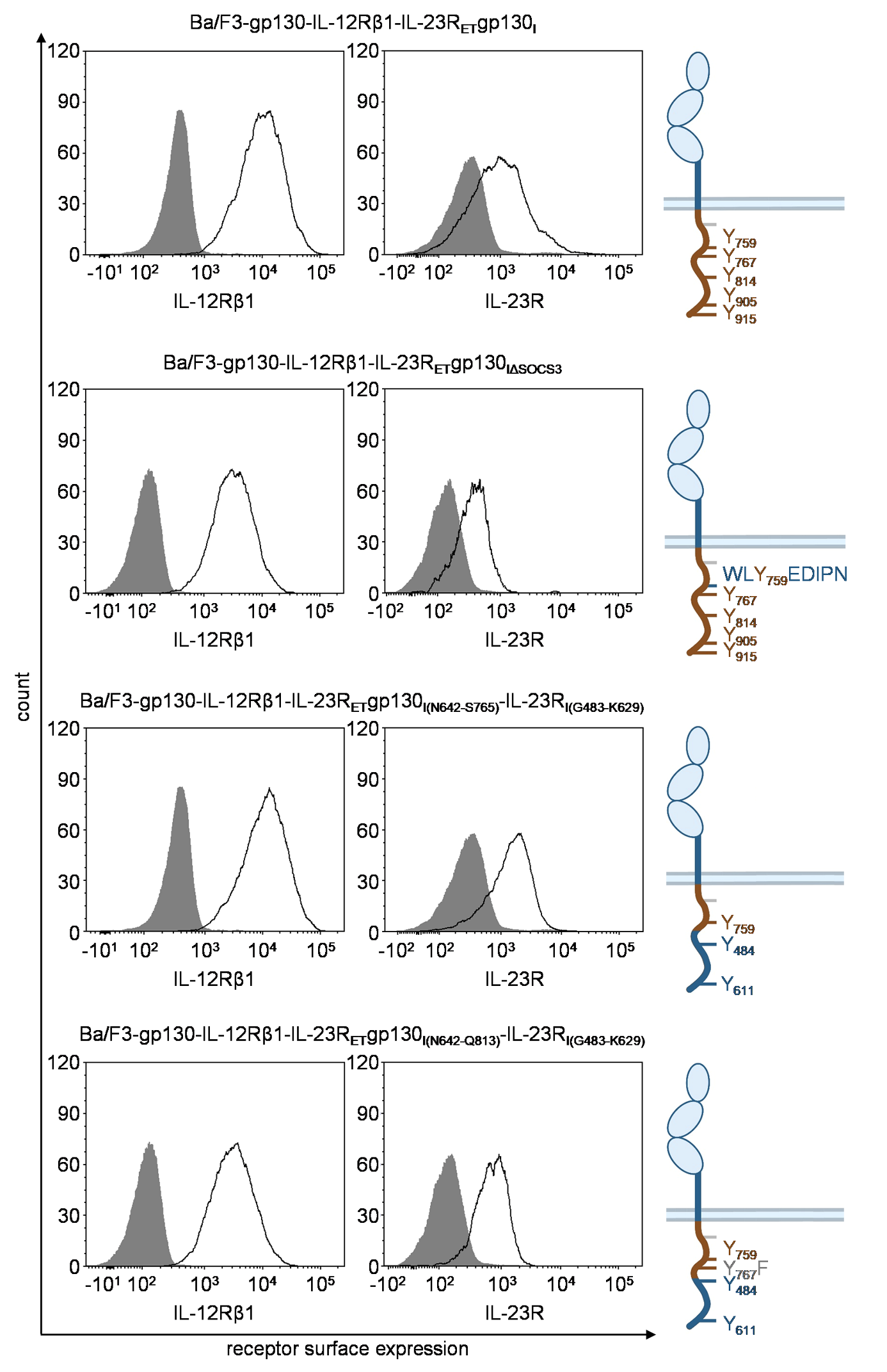


**Additional figure 7: IL-23 receptor surface expression.** Flow cytometry analysis of IL-23 receptors on the surface of Ba/F3-gp130 cells, indicated as solid line. Expression was detected via antibodies against extracellular domains of IL-12Rβ1 (left panel) or IL-23R (right panel). Gray-shade area indicates non-transfected Ba/F3-gp130 cells (negative control).
