# Supplementary material for: Receptor engineering constitutes feedback control and robustness of IL-23R signaling and highlights importance of intracellular cytokine receptor signaling motifs
Source: Cell Commun Signal. 2026 Jan 6;24:51. doi: 10.1186/s12964-025-02593-2 (PMC12849345; doi:10.1186/s12964-025-02593-2)
Supplement: Supplementary file 2 — Supplementary Material 2 [file 12964_2025_2593_MOESM2_ESM.pdf]

Receptor engineering constitutes feedback control and robustness of IL-23R signaling and highlights importance of intracellular cytokine receptor signaling motifs

Leorina Kashtanjeva[1*], Christin Ruhland[1*], Franz Christian Horstmeier[1], Felix Thives-Kurenbach[1], Julia Ettich[1], Giacomo Padrini[1], Sophie Streuber[2], Jürgen Scheller[1], Anna Dittrich[2] and Doreen M. Floss[1#]

[1]Institute of Biochemistry and Molecular Biology II, Medical Faculty and University Hospital Düsseldorf, Heinrich-Heine-University Düsseldorf, Düsseldorf, Germany

[2]Department of Systems Biology, Institute of Biology, Otto-von-Guericke-University Magdeburg, Magdeburg, Germany

[*]These authors contributed equally to this work.

[#]Corresponding author: doreen.floss@hhu.de

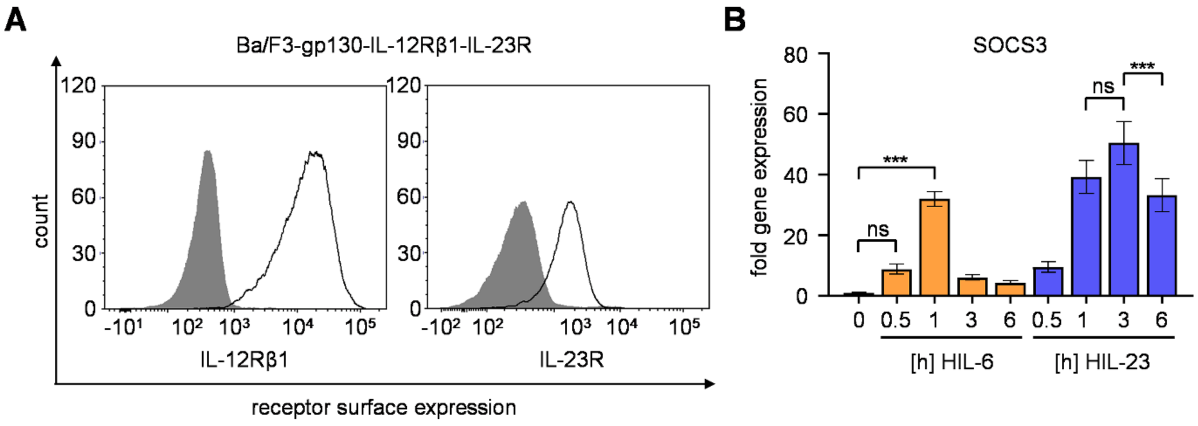

Additional figure 1: Analysis of Ba/F3-gp130-IL-12Rβ1-IL-23R cells with regard to receptor and SOCS3 expression. A) Flow cytometry analysis of IL-23 receptors on the surface of Ba/F3-gp130-IL-12Rβ1-IL-23R cells, indicated as solid line. Expression was detected via antibodies against extracellular domains of IL-12Rβ1 (left panel) or IL-23R (right panel). Gray-shade area indicates non-transfected Ba/F3-gp130 cells (negative control). **B)** Quantification of SOCS3 mRNA expression in stimulated Ba/F3-gp130-IL-12Rβ1-IL-23R cells. The specified times were used for stimulation with HIL-6 (10 ng/ml) or HIL-23 (10 ng/ml). ***$p \leq 0.001$, ns not significant.

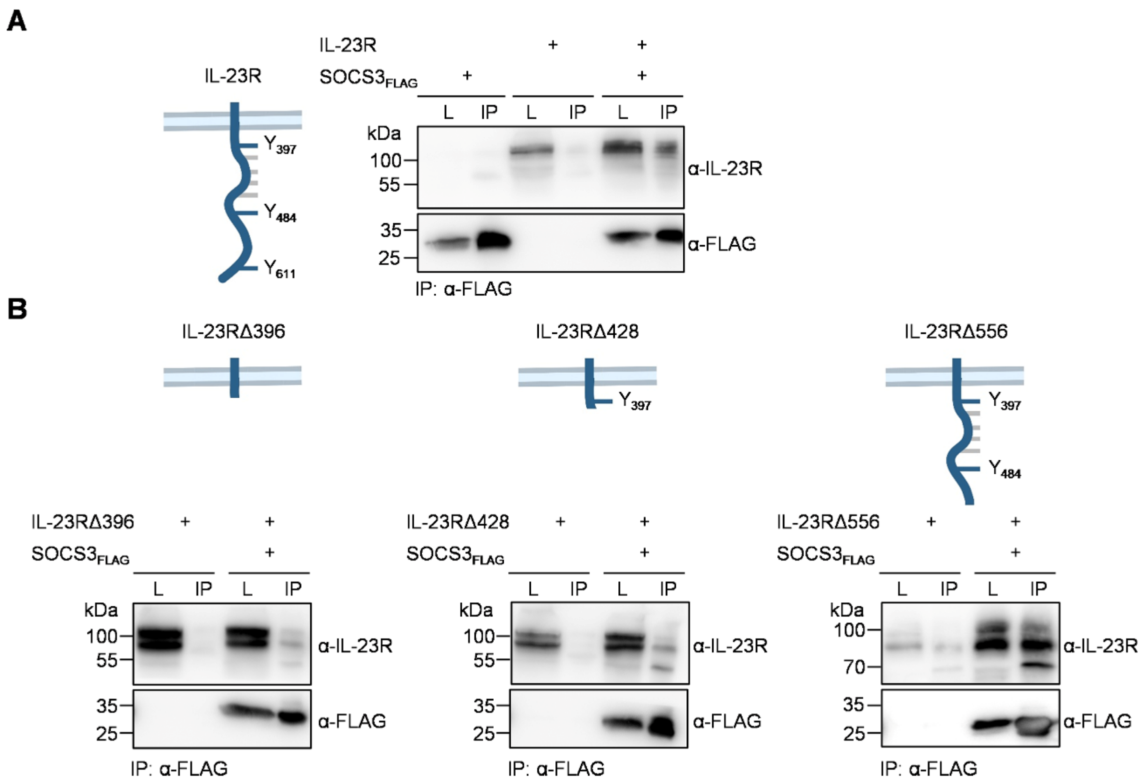

Additional figure 2: SOCS3 interacts with IL-23R. A) Co-IP of FLAG-tagged SOCS3 and full-length IL-23R using ANTI-FLAG® M2 affinity gel. One of two independent experiments is shown. L, lysates; IP, co-immunoprecipitates. **B)** Co-IP of FLAG-tagged SOCS3 and IL-23R deletion variants using ANTI-FLAG® M2 affinity gel. One of two independent experiments is shown. L, lysates; IP, co-immunoprecipitates.

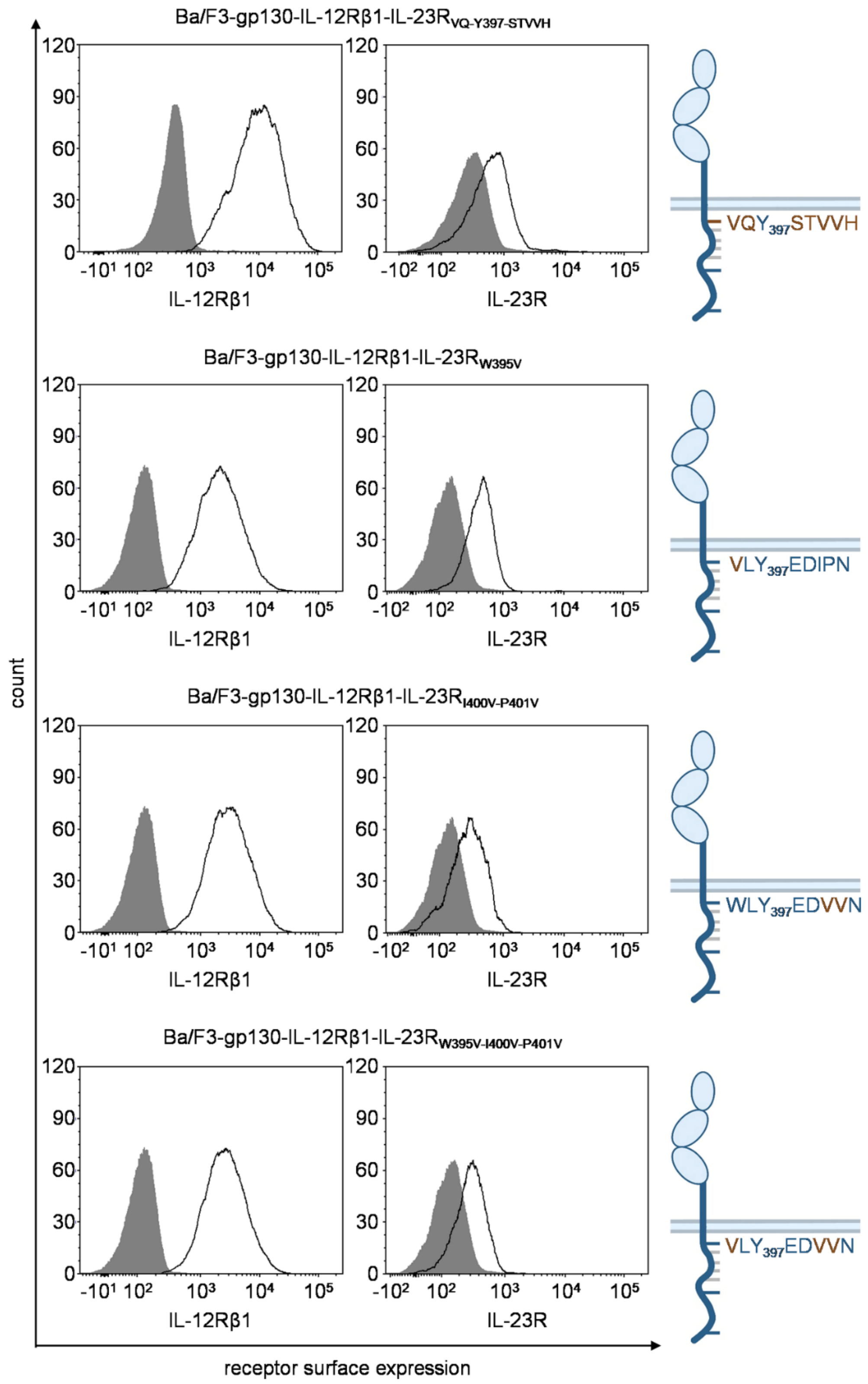

Additional figure 3: IL-23 receptor surface expression. Flow cytometry analysis of IL-23 receptors on the surface of Ba/F3-gp130 cells, indicated as solid line. Expression was detected via antibodies against extracellular domains of IL-12Rβ1 (left panel) or IL-23R (right panel). Gray-shade area indicates non-transfected Ba/F3-gp130 cells (negative control).

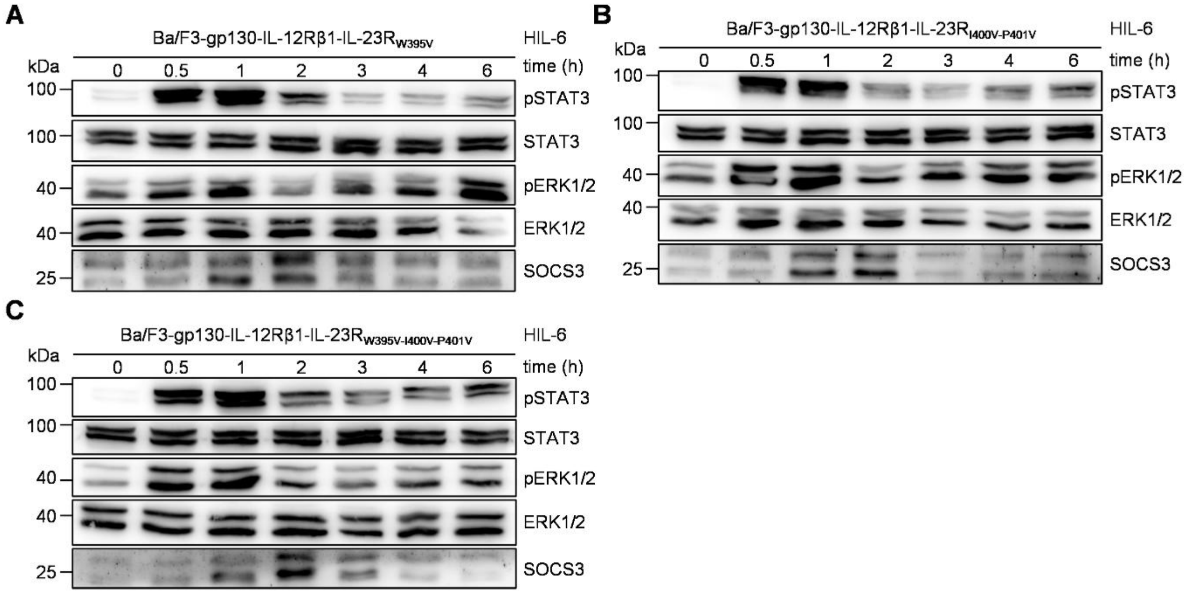

Additional figure 4: SOCS3 induced negative feedback of Ba/F3-gp130 cell lines stimulated with HIL-6. STAT3 and ERK1/2 activation in Ba/F3-gp130 cells expressing IL-12Rβ1 and IL-23R$_{W395V}$ (A), IL-23R$_{I400V-P401V}$ (B) or IL-23R$_{W395V-I400V-P401V}$ (C) treated with HIL-6 (10 ng/ml) for indicated time points or left untreated. Equal amounts of proteins (50 µg/lane) were analyzed via specific antibodies detecting phospho-STAT3 and STAT3, phospho-ERK1/2 and ERK1/2, and SOCS3. Western blot data shows one representative experiment out of three.

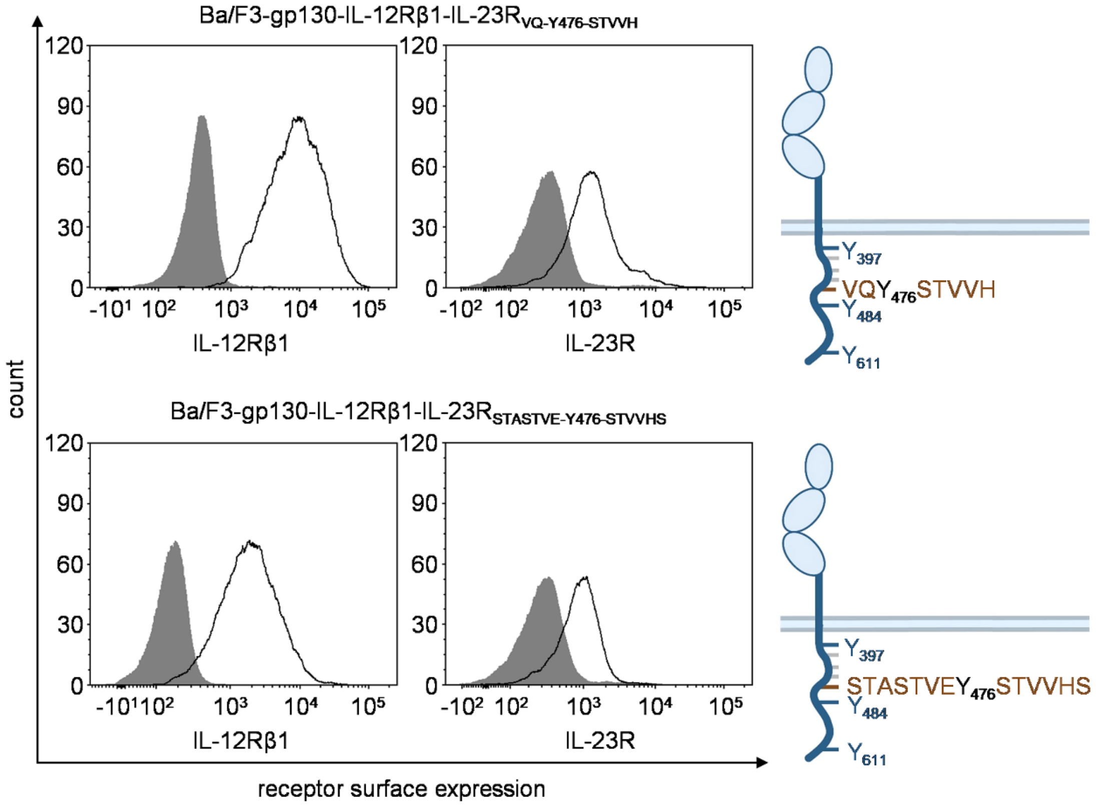

Additional figure 5: IL-23 receptor surface expression. Flow cytometry analysis of IL-23 receptors on the surface of Ba/F3-gp130 cells, indicated as solid line. Expression was detected via antibodies against extracellular domains of IL-12Rβ1 (left panel) or IL-23R (right panel). Gray-shade area indicates non-transfected Ba/F3-gp130 cells (negative control).

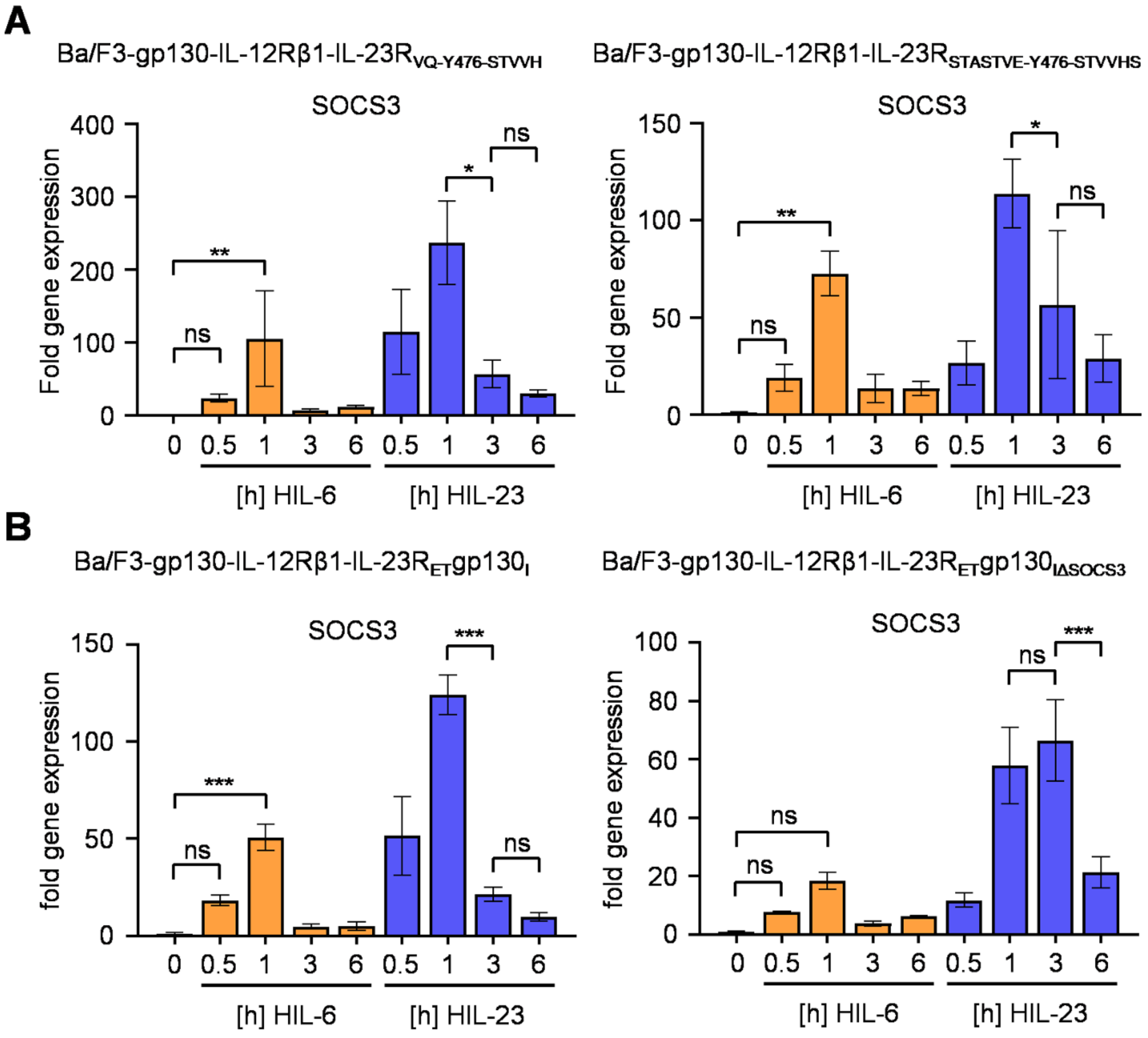

Additional figure 6: Analysis of SOCS3 expression in stimulated Ba/F3-gp130-IL-12Rβ1-IL-23R cells. Quantification of SOCS3 mRNA expression in stimulated Ba/F3-gp130-IL-12Rβ1-IL-23R cells. The specified times were used for stimulation with HIL-6 (10 ng/ml) or HIL-23 (10 ng/ml). **$p \leq 0.01$, **$p \leq 0.05$, ***$p \leq 0.001$, ns not significant.

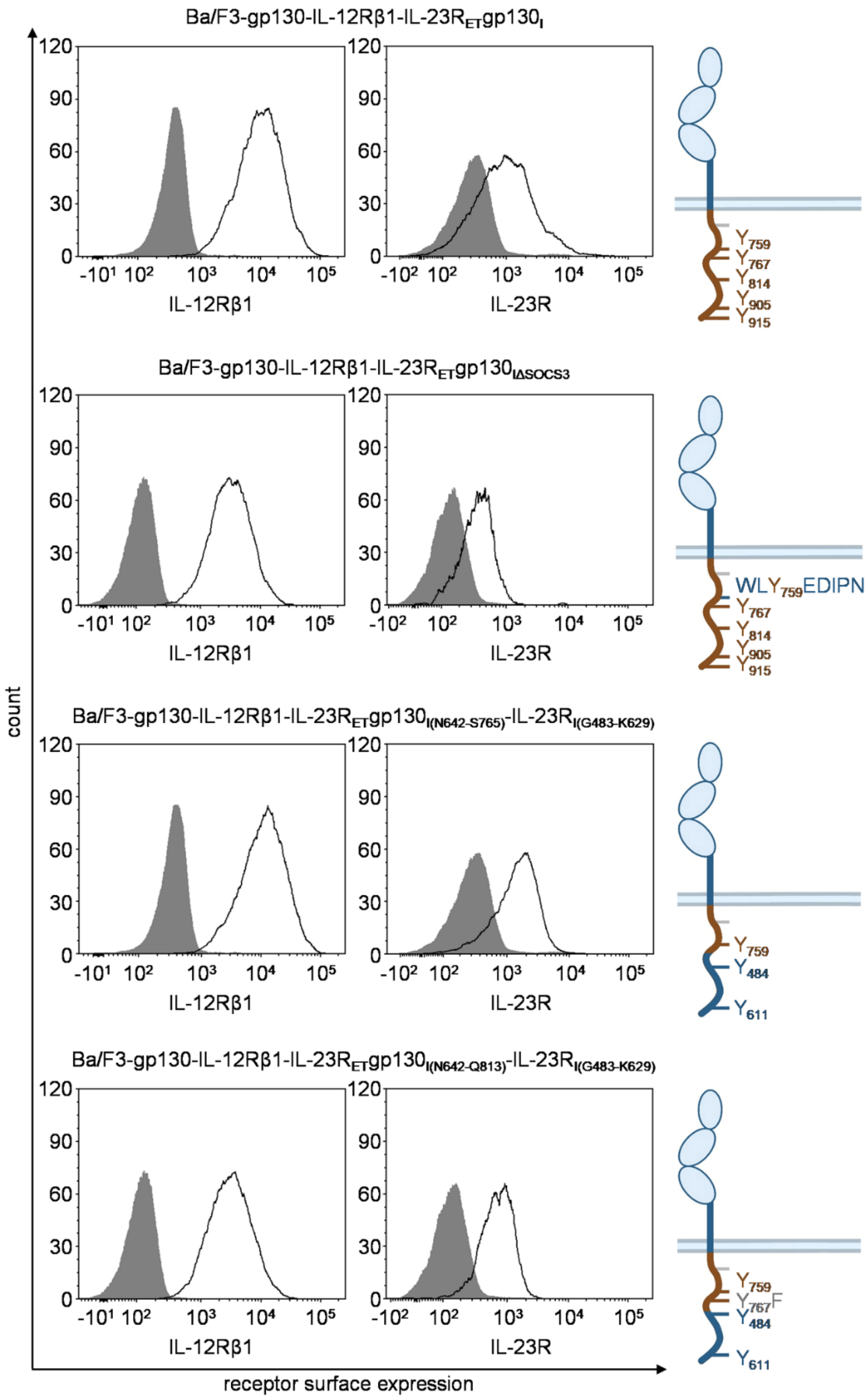

Additional figure 7: IL-23 receptor surface expression. Flow cytometry analysis of IL-23 receptors on the surface of Ba/F3-gp130 cells, indicated as solid line. Expression was detected via antibodies against extracellular domains of IL-12Rβ1 (left panel) or IL-23R (right panel). Gray-shade area indicates non-transfected Ba/F3-gp130 cells (negative control).
